# Modifying Anthocyanins Biosynthesis in Tomato Hairy Roots: A Test Bed for Plant Resistance to Ionizing Radiation and Antioxidant Properties in Space

**DOI:** 10.3389/fpls.2022.830931

**Published:** 2022-02-24

**Authors:** Silvia Massa, Riccardo Pagliarello, Alessia Cemmi, Ilaria Di Sarcina, Aureliano Bombarely, Olivia Costantina Demurtas, Gianfranco Diretto, Francesca Paolini, H. Earl Petzold, Mattijs Bliek, Elisabetta Bennici, Antonella Del Fiore, Patrizia De Rossi, Cornelis Spelt, Ronald Koes, Francesca Quattrocchio, Eugenio Benvenuto

**Affiliations:** ^1^Department for Sustainability, Biotechnology and Agro-Industry Division – Biotec Laboratory, Italian National Agency for New Technologies, Energy and Sustainable Economic Development, Rome, Italy; ^2^Department of Agriculture and Forest Sciences, University of Tuscia, Viterbo, Italy; ^3^Fusion and Nuclear Safety Technologies Department, Italian National Agency for New Technologies, Energy and Sustainable Economic Development, Rome, Italy; ^4^Department of Biosciences, University of Milan, Milan, Italy; ^5^‘Regina Elena’ National Cancer Institute, HPV-UNIT, Department of Research, Advanced Diagnostic and Technological Innovation, Translational Research Functional Departmental Area, Rome, Italy; ^6^School of Plants and Environmental Sciences, Virginia Tech, Blacksburg, VA, United States; ^7^Department of Plant Development and (Epi)Genetics, Swammerdam Institute for Life Sciences, University of Amsterdam, Amsterdam, Netherlands; ^8^Department for Sustainability, Biotechnology and Agro-Industry Division – Agrifood Sustainability, Quality, and Safety Laboratory, Italian National Agency for New Technologies, Energy and Sustainable Economic Development, Rome, Italy; ^9^Energy Efficiency Unit Department – Northern Area Regions Laboratory, Casaccia Research Center, Italian National Agency for New Technologies, Energy and Sustainable Economic Development, Rome, Italy

**Keywords:** MicroTom, hairy root cultures, agrospace, biofortification, anthocyanins, gamma radiation

## Abstract

Gene expression manipulation of specific metabolic pathways can be used to obtain bioaccumulation of valuable molecules and desired quality traits in plants. A single-gene approach to impact different traits would be greatly desirable in agrospace applications, where several aspects of plant physiology can be affected, influencing growth. In this work, MicroTom hairy root cultures expressing a MYB-like transcription factor that regulates the biosynthesis of anthocyanins in *Petunia hybrida* (*PhAN4*), were considered as a testbed for bio-fortified tomato whole plants aimed at agrospace applications. Ectopic expression of *PhAN4* promoted biosynthesis of anthocyanins, allowing to profile 5 major derivatives of delphinidin and petunidin together with pelargonidin and malvidin-based anthocyanins, unusual in tomato. Consistent with *PhAN4* features, transcriptomic profiling indicated upregulation of genes correlated to anthocyanin biosynthesis. Interestingly, a transcriptome reprogramming oriented to positive regulation of cell response to biotic, abiotic, and redox stimuli was evidenced. *PhAN4* hairy root cultures showed the significant capability to counteract reactive oxygen species (ROS) accumulation and protein misfolding upon high-dose gamma irradiation, which is among the most potent pro-oxidant stress that can be encountered in space. These results may have significance in the engineering of whole tomato plants that can benefit space agriculture.

## Introduction

Anthocyanins are valuable water-soluble plant pigments. They accumulate in the vacuole of specialized cells and play a crucial role in pigmentation of flowers and fruits, pollinators attraction, plant-pathogen interaction, protection against ultraviolet (UV) light, and modulation of reactive oxygen species (ROS)-signaling both in reproductive and in vegetative tissues (Brunetti et al., 2013). As plant-derived molecules, anthocyanins are naturally present in the human diet where they are predominantly represented as glycosides, in a multitude of fruits and vegetables, among which berries and grapes have the highest content (Bognar et al., 2013). Anthocyanins act as health-promoting and chronic-diseases-preventing molecules, due to antioxidant, anti-inflammatory, anti-proliferative and anti-neurodegenerative functions (Blesso, 2019; Krga and Milenkovic, 2019; Tian et al., 2019; Bendokas et al., 2020; Kalt et al., 2020). Due to these properties, anthocyanins have increasing applications in the food sector and there is also growing interested in the design of food crops with improved levels and composition of these antioxidant nutraceuticals. In particular, the research involved in the definition of plants intended for ‘agrospace’ applications, will have to tackle the issue to provide fresh and healthy food for space crews in the context of a harmful ionizing irradiated environment, and to cope with cultivation areas subjected to possible biotic contaminations, as well (Amalfitano et al., 2020; Bijlani et al., 2021). Agrospace crops are, therefore, candidates for the application of technologies aiming to improve both their content in antioxidant nutraceuticals and resistance to biotic and abiotic stresses (Zabel et al., 2015, 2016; Massa et al., 2016; Khodadad et al., 2020).

Tomato (*Solanum lycopersicum* L.) is a rich source of bioactive molecules such as carotenoids (in particular, lycopene), polyphenols and flavonoids, ascorbic acid, and other vitamins (Gerszberg et al., 2015; Martí et al., 2016). However, anthocyanins are poorly accumulated in cultivated tomatoes and even the fruits of cultivar harboring natural mutants for the *Abg* (*Aubergine*), *Aft* (*Anthocyanin fruit*), and *Atv* (*Atroviolaceum*) loci, only contain these molecules in the peel (Mes et al., 2008; Gonzali et al., 2009; Povero et al., 2011). Accumulation in fruit flesh and other organs upon genetic engineering indicates that tomatoes can be manipulated to this end (Zhang et al., 2014; Lloyd et al., 2017). By breeding, tomato lines were generated that combine the dominant *Atv* allele with *Aft* or *Abg*. These lines accumulated up to 0.1% (in fresh weight) of the anthocyanin petunidin-3-(p-coumaryl)-rutinoside-5-glucoside in the fruit epidermis (Mes et al., 2008; Povero et al., 2011). Gene and pathway engineering are powerful approaches to enhance the biosynthesis of anthocyanins in plants, and they have been successfully applied in food staples (Garg et al., 2018). Transcription factors, regulating the expression of structural biosynthetic genes, control the activity of the anthocyanin pathway in all plant species (Gonzalez et al., 2008). In particular, proteins belonging to specific clades of R2R3-MYB, bHLH, WDR, and WRKY have been shown to regulate anthocyanins biosynthesis combined in the MBWW transcription complex, as shown for a multitude of plant species among which tomato (Ramsay and Glover, 2005; Zhang et al., 2014; Gao et al., 2018). The combined expression of a MYB and bHLH regulators of the anthocyanin pathway from *Antirrhinum majus* (*Delila* and *Rosea1*, respectively) under a fruit-specific promoter, resulted in the production of anthocyanins in fruits peel and flesh of tomato (Butelli et al., 2008). Studies have demonstrated that pathway engineering approaches implying the sole use of MYB factors belonging to the SG6 clade are sufficient to restore the biosynthesis of anthocyanins by promotion of the transcription of their bHLH partners and, therefore, by reconstituting the MBWW (Mehrtens et al., 2005; Takos et al., 2006; Zhang et al., 2019). The tomato *ANT1* gene encodes a MYB transcription factor belonging to the SG6 clade, highly homologous to the *Antirrhinum Rosea*. It has been demonstrated that ectopic expression of *ANT1* from a tomato wild relative (*S. chilense*), induces purple spotting on the epidermis of tomatoes (Mathews et al., 2003; Schreiber et al., 2012). The 35S promoter-driven expression of either the *Solanum lycopersicum ANT1* or *AN2* (another SG6 MYB), has been shown to induce anthocyanins production in the flesh and peel of the fruit and different organs of tomato plants (Kiferle et al., 2015). Upon overexpression of *SlAN2*, together with anthocyanins accumulation in fruits, flower organs, and vegetative parts, an enhancement of the emission of volatile molecules contributing to the aroma of fruits was found, as well (Jian et al., 2019). In addition, *SlAN2* has been related to the variation of levels of specialized metabolites other than anthocyanins, and of fruit softening (Meng et al., 2015). These findings seem to confirm that MYB transcription factors of the SG6 clade can regulate various, sometimes unrelated, processes in tomatoes, as well (Stracke et al., 2001; Zimmermann et al., 2004; Zhang et al., 2019). Therefore, a SG6 MYB-based approach may be considered suitable to affect multiple pathways in tomatoes.

The *Anthocyanin4* gene of *Petunia hybrida* (*PhAN4*) is a SG6 member of a small family of genes encoding very similar MYBs phylogenetically related to the snapdragon *AmROSEA*, the tomato *SlANT1*, and other anthocyanin-regulating MYBs from a multitude of plant species. All these petunia MYBs are involved in the induction of anthocyanins accumulation in different plant parts and response to different stimuli (Povero, 2011). In the present study, we performed *Agrobacterium rhizogenes*-mediated transfer of a construct for the expression of *PhAN4* into the miniature tomato genotype MicroTom to generate hairy root cultures (HRCs). HRCs were intended as a testbed for whole plant engineering strategies able to improve traits for space cultivation. We previously reported about improved *in vivo* response to space-mimicking conditions (i.e., static magnetic fields and X and gamma rays) of *PhAN4*-engineered HRC (Villani et al., 2017; Desiderio et al., 2019), confirming that this plant-based expression system, used over the last 30 years to produce various specialized metabolites and recombinant proteins of pharmaceutical value (Gutierrez-Valdes et al., 2020; Häkkinen et al., 2020), is useful in studies on the adaptation of plants to extraterrestrial conditions, as well. Recently, HRCs served in several plant species as handier and faster biotechnology tools, compared to whole plant transformation, to gain biological insights in gene function, spatial and temporal gene expression studies, and signaling pathways in plant cell response to a changing environment (Ron et al., 2014).

Anthocyanin biosynthesis engineering in HRCs was reported in a few species (Sharma et al., 2013; Li et al., 2016; Thwe et al., 2016; Hou et al., 2017), and, to date, never in tomatoes. In this study, *PhAN4* gene expression resulted in anthocyanins accumulation in tomato HRCs. Transcript profiling showed that several genes encoding enzymes and transcription factors involved in anthocyanins biosynthesis were upregulated. Interestingly, also genes correlated to cell response to biotic and abiotic stress, including redox stimuli, resulted in transcriptionally upregulated.

In addition, we report about the antioxidant properties and diminished generation of ROS in HRCs expressing *PhAN4* exposed to ionizing gamma radiation. *PhAN4* HRCs were endowed with a ninefold enhanced antioxidant capacity *per se* compared to controls by 2,2-diphenyl-1-picrylhydrazyl (DPPH) assay. Furthermore, ROS accumulation was counteracted after gamma radiation, as shown by Electron Spin Resonance (ESR) Spectroscopy. Both UV-VIS spectra and photoluminescence analysis demonstrated that polyphenols content and stability of soluble protein folding were not significantly affected by high dose gamma irradiation in *PhAN4*-engineered HRCs compared to control.

In conclusion, MicroTom HRCs represented a simplified model that allowed to rapidly test *PhAN4* expression effects on tomato cells, possibly opening the way to the application of the strategy to the engineering of whole plants intended for cultivation in harsh environments like future space outposts.

## Materials and Methods

### Gene and Constructs

*Anthocyanin4* (*PhAN4*) complementary DNA (cDNA) from petals of *Petunia* × hybrida cultivar Violet 30 (GenBank: HQ428105.1) was amplified with primers containing AttB sites and recombined into pDONR221 (RU Ghent) to produce an entry clone. This was then recombined with pKGW,0 (RU Ghent) to produce the 35S:AN4 construct and in pK7FWG2 (RU Ghent) to yield the 35S:GFP-*PhAN4* construct (where the *GFP* gene fusion was adopted to possibly stabilize the PhAN4 transcription factor).

### Hairy Root Cultures Generation

*Solanum lycopersicum* (cv. MicroTom) clonal hairy root lines were obtained from wild-type leaf explants by infection with *A. rhizogenes* A4 (ATCC, 43057™) harboring either the 35S:*PhAN4* or the 35S:GFP-*PhAN4* or no additional construct. Bacteria were grown in a YEB medium with 50 μg/ml rifampicin and 50 μg/ml kanamycin at 28°C and 220 rpm to OD600 = 0.6. Bacteria were centrifuged at 3,000 × *g* for 15 min and resuspended at OD_600_ = 1 in Murashige and Skoog medium (MS, Duchefa) with 30 g/l sucrose and 200 μM acetosyringone, pH 5.8. Leaves from 3-week-old MicroTom plants were harvested, sterilized in 0.1% (v/v) sodium hypochlorite solution for 15 min, and aseptically cut into explants of 1 cm × 1 cm. Explants were immersed in the recombinant *A. rhizogenes* suspension for 15 min, in a rotary shaker at the minimum speed, and in the dark. Explants were dried onto sterilized tissue paper and transferred on their adaxial side, on MS agar medium co-culture plates with 100 μM acetosyringone and incubated in the dark for 4 days. Explants were then blotted and transferred to MS medium supplemented with 250 μg/ml cefotaxime (Cef) at 25°C. Fresh growing hairy roots were obtained after 8–10 days. Emerging roots of 1 cm in length were excised and transferred to new plates. *A. rhizogenes* was eliminated with decreasing Cef concentrations (0.25, 0.125, and 0.05 μg/ml) until no antibiotic was added. HRCs were screened for pigmentation under a dissecting microscope. Growth was estimated by the increase in fresh weight at different time points after subculture over a 28-day culture period recorded for three biological replicates for chosen hairy root clones. Hairy root biomass harvested for analysis was carefully handled, pulverized in liquid nitrogen, and immediately stored at −80°C. For metabolite content and antioxidant properties analysis, HRCs were lyophilized in a freeze-dry system (FreeZone Labconco, Kansas City, MO, United States).

### Polymerase Chain Reaction Assays

Standard polymerase chain reaction (PCR) assays were performed on genomic DNA of kanamycin-resistant hairy root clones (extracted with NucleoSpin Plant II Kit; Macherey-Nagel; Duren, Germany) with primers specific for *PhAN4*, *rol B, rol C*, *virC1*, respectively (Supplementary Table 1), to select hairy root lines carrying *PhAN4* transgene clean from *A. rhizogenes* in the tissue culture. In selected HRC clones, SYBR Green real-time PCR was used to determine the *PhAN4* copy number. The tomato actin 41 gene (NCBI Reference Sequence: NM_001330119.1) served as an endogenous gene reference. For qPCR (i-Cycler iQ detection system; BioRad Laboratories Inc., Milan, Italy) Kapa SYBR Fast 2× qPCR Master Mix (KAPA Biosystems, Milano, Italy) was used, according to the manufacturer’s instructions. Samples were amplified at 95°C for 3 min, followed by 40 cycles of denaturation at 95°C for 15 s, annealing, and extension at 60°C for 30 s.

Total RNA was isolated using the RNeasy Plant Mini Kit (Qiagen; Valencia, CA, United States) and then treated with amplification grade DNaseI (Invitrogen, Cambridge, MA, United States). cDNA was synthesized using the iScript™ cDNA Synthesis Kit (BioRad Laboratories Inc., Milan, Italy) and used as a template for real time-PCR analysis (Kapa SYBR Fast 2× qPCR Master Mix; KAPA Biosystems, Milan, Italy) in iCycler iQ detection system (BioRad Laboratories Inc., Milan, Italy). The actin 41 gene was used as the reference gene. Primers are listed in Supplementary Table 1. Relative gene expression levels were obtained using the 2^–ΔCT^ formula (Livak and Schmittgen, 2001).

### Phenylpropanoids Identification

Anthocyanin profile was carried out on representative *PhAN4* and GFP-*PhAN4* HRCs by liquid chromatography coupled to high-resolution mass spectrometry (LC-HRMS) as reported before (Diretto et al., 2019; Carmona et al., 2021) with slight modifications. Briefly, 3 mg (dried weight) of ground hairy roots were re-suspended in 600 μl of 85:15 MeOH: 1N HCl, vortexed, shaken in Mixer Mill (MM) for 15′ at 20 Hz frequency and gently mixed at 4°C O.N. Samples were then centrifuged at 20,000 × *g* for 20 min, the supernatant recovered, completely dried and re-suspended in 600 μl of spiked (with 0.5 μg/ml formononetin, as internal standard) 75% MeOH + 0.1% formic acid. Samples were then centrifuged 10 min at 20,000 × *g* at RT, and the supernatant was transferred to HPLC vials for MS analysis with a Q-Exactive mass spectrometer (Thermo Fisher Scientific, Cambridge, MA, United States), coupled to a HPLC system equipped with a photodiode array detector (Dionex, Califiornia, United States). LC separation of anthocyanins was performed injecting 5 μl of sample on a C18 Luna reverse-phase column (100 × 2.1 mm, 2.5 μm; Phenomenex, Torrance, CA, United States), using as mobile phase water + 0.1% formic acid (A) and acetonitrile + 0.1% formic acid (B) at a total flow rate of 250 μl/min. The separation was developed using 5% B for 0.5 min, followed by a 24 min linear gradient to 75% B. The ionization was performed using heated electrospray ionization (HESI) source, with nitrogen used as sheath and auxiliary gas, and set to 35 and 10 units, respectively. The vaporizer temperature was 250°C, the capillary temperature was 30°C, the spray voltage was set to 3.5 kV, the probe heater temperature was 390°C, and the S-lens RF level was set at 50. The acquisition was performed in the mass range 110/1,600 m/z both in positive and in negative ion mode with the following parameters: resolution 70,000, microscan 1, AGC target 1e6, maximum injection time 50. UV-VIS detection was continuous from 220 to 700 nm. All solvents used were LC-MS grade (Merck Millipore, Burlington, MA, United States). Identification was achieved based on accurate masses and by comparison with authentic reference substances. The ion peak areas were normalized to the ion peak area of the internal standard (formononetin).

Total anthocyanins content was measured by spectrophotometric analysis, as described in the study of Brito et al. (2014), using the extinction coefficient of the most abundant anthocyanin (petunidin-3-(p-coumaroyl)-rutinoside-5-glucoside).

### Determination of the Total Phenolics Accumulation Level

Total phenolic content of representative *PhAN4* and GFP-*PhAN4* MicroTom HRC was estimated by colorimetric assay with modified (Inglett et al., 2010) Folin–Ciocalteu reagent (Merk, Germany) (Şensoy et al., 2006). Briefly, 4.25 ml of de-ionized water was mixed with 0.25 ml of ethanolic extract diluted 1:5 with 80% (v/v) ethanol and 0.25 ml of Folin-Ciocalteu reagent. After 7 min incubation in the dark at room temperature, 0.5 ml of saturated sodium carbonate solution (20%) was added, and the mixture was incubated for 40 min in the dark at room temperature. Absorbance was measured at 725 nm using a UV-VIS spectrophotometer (PerkinElmer, Waltham, MA, United States). A standard curve was prepared with gallic acid, as the reference standard. Final values were obtained by interpolating the absorbance values recorded for tomato hairy root extracts with the gallic acid calibration curve. The total phenolic content was expressed as μg of gallic acid equivalents (GAE)/g of dry weight. Each analysis consisted of triplicate measurements of each sample and data were averaged over the three measurements.

### 1,1-Diphenyl-2-Picrylhydrazyl Radical Scavenging Activity Assay

Phenolic compounds accumulating in representative *PhAN4* and GFP-*PhAN4* HRCs were extracted (Morishita et al., 2007) to apply the DPPH free radical scavenging method in order to establish their antioxidant properties. Ground, freeze-dried tomato HRC (30 mg) were extracted with 0.6 ml EtOH 80% (v/v), shaken in a water bath at 80°C for 40 min, and then centrifuged at 3,000 rpm for 15 min. The recovered supernatant was filtered through polytetrafluoroethylene membrane (0.45 μm) and stored at −20°C. The antioxidant capacity of the ethanolic extracts was spectrophotometrically tested by DPPH (1,1-diphenyl-2-picrylhydrazyl) (Li et al., 2010), with some modifications. Briefly, 0.25 ml of diluted ethanolic extract was added to 2.9 ml of 0.06 mM DPPH working solution. The mixture was shaken and allowed to stand at room temperature, in the dark, for 30 min. Absorbance was measured at 515 nm using a UV-VIS spectrophotometer. Lower absorbance values of the reaction mixture indicated higher free radical scavenging activity. The inhibition of free radical DPPH was expressed as DPPH scavenging effect (% inhibition) I% = {(A0 − A1)/A0) × 100}, where A1 and A0 are the absorbance values of blank and of tested samples, respectively. Trolox (vitamin E equivalent antioxidant) was used as the reference standard. Each analysis consisted of triplicate measurements of each sample and data were averaged over the three measurements.

### Complementary DNA *L*ibrary *C*onstruction and *S*equencing for *T*ranscriptomic *A*nalysis

Total RNA from wild type and *PhAN4*-1 HRC was extracted using the RNeasy Plant Mini Kit (Qiagen; Valencia, CA, United States). RNA was quantified using the Qubit ^®^ fluorometer (Thermo Fisher Scientific, Waltham, MA, United States) and assayed through Agilent 2100 Bioanalyzer ^®^ (Agilent Technologies, Santa Clara, CA, United States) for quality and Integrity Number (RIN) evaluation. Samples with 8 ≤ RIN ≤ 10 were considered. For each sample, equal amounts of RNA (2 μg) extracted from three biological replicates were pooled for cDNA library construction. First, the poly-A mRNA in the total RNA was pulled down using poly-T oligo-attached magnetic beads. After purification, the mRNA was fragmented at 95°C for 2 min along with RT primer and first-strand buffer. The fragmented RNA was used for the synthesis of the first-strand cDNA by adding DTT, dNTPs, Rnase Inhibitor, and SMARTScribe. All chemicals were incubated for 2 h at 42°C. This was followed by second-strand cDNA synthesis using template-switching oligonucleotide and an additional 1 μl of SMARTScribe. Subsequently, the cDNAs were purified using two rounds of AMPure beads. The samples were enriched by using PCR to create the final library. A final fragment size purification step was performed using the Blue Pippin system selecting fragments between 250 and 500 bp. The libraries were confirmed by electrophoresis on a 1% TAE agarose gel and the Agilent BioAnalyzer 2100 ^®^ after purification by AMPure bead. The average read size was estimated at 250–350 bp. The libraries were sequenced at Novogene Corporation using two lanes of an Illumina HiSeq4000 system with a pair-end run of 2 × 150 bp (Illumina, San Diego, CA, United States).

### Sequencing Read Mapping and Identification of Differentially Expressed Genes

Raw RNA-seq libraries were analyzed according to the bioinformatic in-house pipeline (University of Amsterdam, Netherlands)^1^. The raw reads (in FASTQ format) generated from sequencing were cleaned using Trimmomatic version 0.36 (Institut Pasteur, France) (Cock et al., 2009) by removing adaptor-polluted reads, reads with unknown sequences “N” accounting for more than 5% and low-quality reads (with a mass value less than 10 and proportion of a total number of bases in the reads greater than 20%). The clean reads were mapped to the *S. lycopersicum* reference genome sequence version ITAG4 (Consortium et al., 2012) downloaded from the Sol Genomics Network database (Fernandez-Pozo et al., 2015). Two programs were used for this purpose: Hisat2 version 2.1 (Institut Pasteur, France) (Kim et al., 2015) and STAR version 2.5.2b (Institut Pasteur, France) (Dobin et al., 2013). The mapping results were compared with the Picard Tools CollectAlignmentSummaryMetrics version 1.138 (Broad Institute, Cambridge, MA, United States)^2^. The STAR mapping results were selected for further analysis. BAM files were transformed to a subread matrix file using the Rsubread version 1.34.4 R package (Bioconductor, open source) (Liao et al., 2019). Differential expression analysis was performed with the DESeq2 R package version 1.22.2 (Bioconductor, open source) (Love et al., 2014). Genes with an adjusted *P*-value of ≤0.001 and a log2 fold change of ≥2 were defined as differentially expressed (Benjamini and Hochberg, 1995).

### Functional Annotation and Enrichment Pathway Analysis of Differentially Expressed Genes and Identification of Tomato Genes

The list of DEGs (Cluster 1 – *PhAN4*/WT < 1 and Cluster 2 – *PhAN4*/WT > 1) was analyzed using the g:Profiler (Reimand et al., 2007) with the default parameters using Organism *Solanum lycopersicum*. No terms were statistically significant under the “Measure underrepresentation” option. The results were exported as CSV and uploaded into R Studio where they were plotted with ggplot2^3^. The gene functional annotation was performed by sequence homology search with different protein data sets using BLASTP and Protein domains search using InterPro Scan.

### Measurements of Tomato Hairy Root Cultures pH

Measurement of both control and PhAN4 HRCs pH was accomplished as described by Verweij et al. (2008). Briefly, 10 mg of hairy root material were ground in 2 ml distilled water and immediately measured with a pH electrode (edge ^®^ Multiparameter pH Meter – Hanna Instruments, Italy).

### Gamma Irradiation Tests

Irradiation tests were performed at the Calliope facility, a pool-type irradiation plant equipped with a ^60^Co gamma source in a high volume (7 m × 6 m × 3.9 m) shielded cell at ENEA (Casaccia Research Centre, Rome, Italy). The source emits radiations consisting of two gamma photons with a mean energy of 1.25 MeV (Baccaro et al., 2019). Fricke dosimetric system was employed for the determination of the absorbed dose during the irradiation tests. HRCs-derived samples were irradiated at room temperature, at three different absorbed doses (0.5, 1, and 2 kGy), and a dose rate of 1.8 kGywater/h.

### Electron Spin Resonance Spectroscopy Before and After Gamma Irradiation

The molecular species accumulating upon the ectopic expression of *PhAN4* were investigated for possible efficient maintenance of the ability to counteract the generation of reactive oxygen species upon strong ROS inducers such as ionizing radiations. Gamma rays were used to generate peroxyl radicals (which are proportional to the number of paramagnetic species present in the samples) in lyophilized HRC powder. We explored ROS formation before and after 0.5, 1, and 2 kGy absorbed dose in AN4-1 and control HRCs sample sets by ESR Spectroscopy measurements. Not irradiated sample sets were used as references. Each set consisted of two replicates. For each ESR analysis, 8 ± 0.1 mg of HRC lyophilized powder was split into two PT-Capillaries (NOX-A.8.1-PT NOXYGEN, Holland) that were then inserted in a conventional quartz sample tube (o.d./i.d. of 4/3 mm) closed by a plastic lid. Irradiated samples were analyzed straight after the end of irradiation and ESR signals and were normalized to the sample mass. ESR measurements were acquired using an ESR e-scan spectrometer (Bruker, Billerica, MA, United States) operating in the X-band frequency (9.4 GHz) with a field modulation frequency of 86 kHz and modulation amplitude of 5.152 G. The ESR spectra were recorded at a central magnetic field of 3466 Gauss with a sweep width of 160 G, microwave power of 0.14 mW, microwave frequency of 9.75 GHz. The ESR spectra reported in this work derived from the accumulation of four scans. Bruker WinEPR data processing software (Bruker, Billerica, MA, United States) was used for data elaboration.

### Ultraviolet-Visible Absorbance Spectra Analysis Before and After Gamma Irradiation

Ultraviolet-visible (UV-VIS) spectra were obtained from crude extracts of AN4-1 and control HRCs. Briefly, lyophilized HRCs were ground in liquid nitrogen and the resulting powder was finely homogenized using an Ultraturrax homogenizer (IKA, Germany) in water:HCl (100:1, v/v). Samples were incubated at 500 rpm for 1 h at R.T., clarified by centrifugation at 11,000 × *g* for 30 min. The resulting supernatants were examined at 280–600 nm, by a UV-spectrophotometer (Lambda 950, Perkin Elmer, Waltham, MA, United States) at R.T. with a slit width of 2 nm, using a 10 mm cell.

### Photoluminescence Analysis Before and After Gamma Irradiation

Samples were finely ground in liquid nitrogen with mortar and pestle, resuspended, and homogenized in phosphate-buffered saline pH 7.2 (PBS, 1:3 w/v) containing a protease inhibitor cocktail (Complete™; Roche, Mannheim, Germany) to extract soluble proteins. Photoluminescence emission spectra of extracts were determined before and straight after 2 kGy absorbed dose in AN4-1 and control HRC dried biomass sample sets. Two replicates per set were poured into quartz cuvettes with an optical path length of 1 cm (104F-QS, Hellma, Germany). The emission spectra were recorded using the Edinburgh Instruments FS 5 spectrometer in the range 300–800 nm with 280 nm excitation wavelength. The recorded spectra were mass-normalized and corrected for background scattering (reference: extraction buffer).

### Statistical Analysis

All data (HRCs growth, *PhAN4* gene expression, total phenolic content, DPPH antioxidant capacity, total anthocyanin content) were subjected to one-way ANOVA with Tukey’s post-test to determine the differences in average of all tested parameters ± *SD*. A *p*-value less than 0.05 was considered statistically significant. GraphPad Prism version 8.0.2 for Windows (GraphPad Software, San Diego, CA, United States) was used for graphical and statistical data processing.

## Results

### Hairy Roots Generation and Screening

**FIGURE 1:**
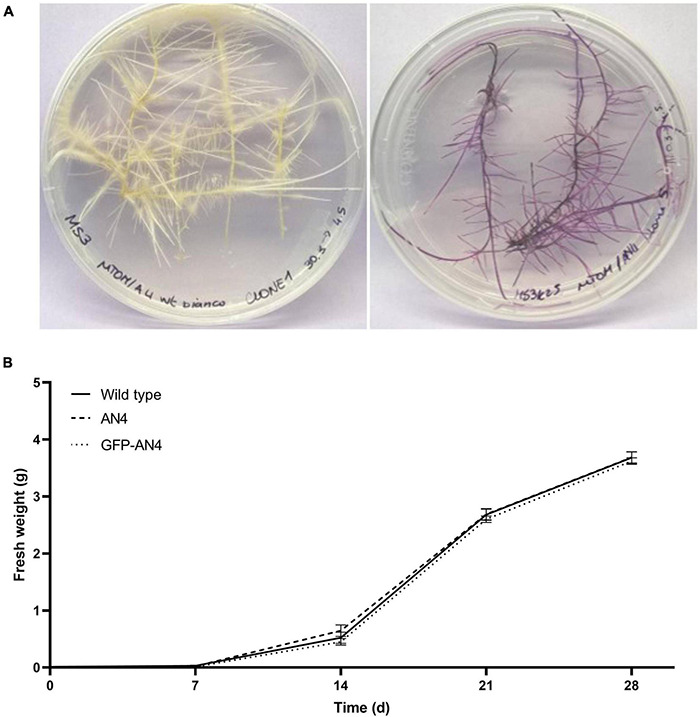
Control (left) and transformed (35S:AN4, right) MicroTom hairy roots **(A)**. Hairy root culture (HRC) growth estimation by an increase in fresh weight along 28 days of cultures in MS medium (one-way ANOVA analysis of variance with Tukey’s *post-hoc* test) by GraphPad Prism **(B)**.

Both 35S:*PhAN4* and 35S:GFP-*PhAN4* constructs were independently transferred into the miniature tomato genotype MicroTom to generate hairy root cultures. Control HRCs were obtained by transformation with *A. rhizogenes* not containing *PhAN4*. HRCs were collected from independent explants. While control HRCs grew as unpigmented organ cultures (Figure 1A, left), HRCs generated by 35S:*PhAN4* and 35S:GFP-*PhAN4* showed purple pigmentation (Figure 1A, right) that may vary among clones. Purple pigmentation was present on primary and secondary branches and was maintained on kanamycin selection (Supplementary Figure 1). HRCs showed typical abundant secondary branching. Three clones for each construct were selected and further analyzed. No statistical difference was found in the growth rate of PhAN4, GFP-PhAN4, and control HRCs (Figure 1B).

Polymerase chain reaction (PCR) screening showed amplification of the expected fragments from genomic DNA, confirming integration of the necessary root-inducing genes from *A. rhizogenes* and of the *PhAN4* transgene (Supplementary Figure 2A). No transgene loss was observed over time (Supplementary Figure 2B). Control HRCs were negative for *PhAN4* amplification, as expected. Integration of *PhAN4* was estimated at copy numbers ranging from 4 to 8 copies, depending on the selected clone analyzed (Supplementary Figure 3). *PhAN4* transcripts were detected in both HRCs harboring either 35S:*PhAN4* or 35S:GFP-*PhAN4* constructs, while they were absent in the control, as expected (Supplementary Figure 3).

### Liquid Chromatography Coupled to High-Resolution Mass Spectrometry Analysis of Phenylpropanoids

**FIGURE 2:**
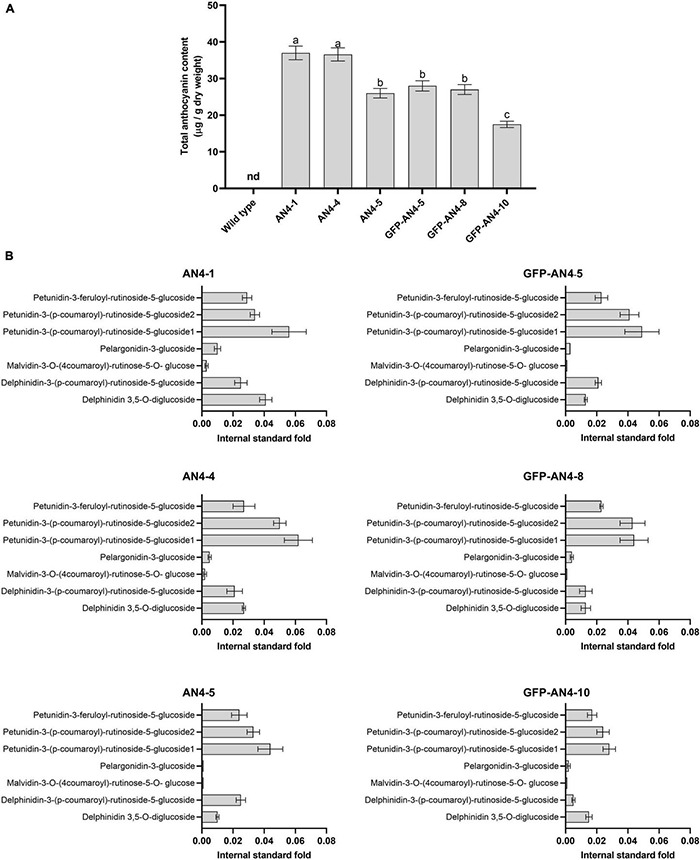
Anthocyanins in PhAN4 HRC clones. Total anthocyanins content **(A)** and amount of specific anthocyanins **(B)** of six representative PhAN4-expressing hairy root clones. Columns report average values ± *SD* (*n* = 3).

Total anthocyanins content was measured. AN4-1 and AN4-4 HRCs showed the highest anthocyanins concentrations, equal to 37 and 36.6 μg/g_dried weight_ (Figure 2A), respectively. The anthocyanins profile was determined by LC-HRMS and compared to control HRCs. Identification was achieved by m/z ion reconstruction starting with the aglycon (delphinidin, petunidin, pelargonidin, malvidin), followed by the recognition of all the conjugated sugar and phenolic moieties. Subsequently, absolute quantification was performed as previously described (Diretto et al., 2019; Carmona et al., 2021) and by interpolating anthocyanin signal intensities in the roots compared to the ones of external calibration curves of the Pelargonidin-3-glucoside and Delphinidin 3,5-*O*-diglucoside standards. In our experimental conditions, anthocyanins were virtually undetectable in control HRCs. Petunidin-3-(p-coumaroyl)-rutinoside-5-glucoside1 and Delphinidin 3,5-*O*-diglucoside were the most abundant anthocyanins in the *PhAN4* roots, followed by a second group including Petunidin-3-(p-coumaroyl)-rutinoside-5-glucoside2, Petunidin-3-feruloyl-rutinoside-5-glucoside and Delphinidin-3-(p-coumaroyl)-rutinoside-5-glucoside. Two additional anthocyanins, Pelargonidin-3-glucoside and Malvidin-3-*O*-(4”’coumaroyl)-rutinose-5-*O*-glucose, were detected, although at low levels (Figure 2B; Su et al., 2016). All lines show about the same relative amount of the different anthocyanin species.

**FIGURE 3:**
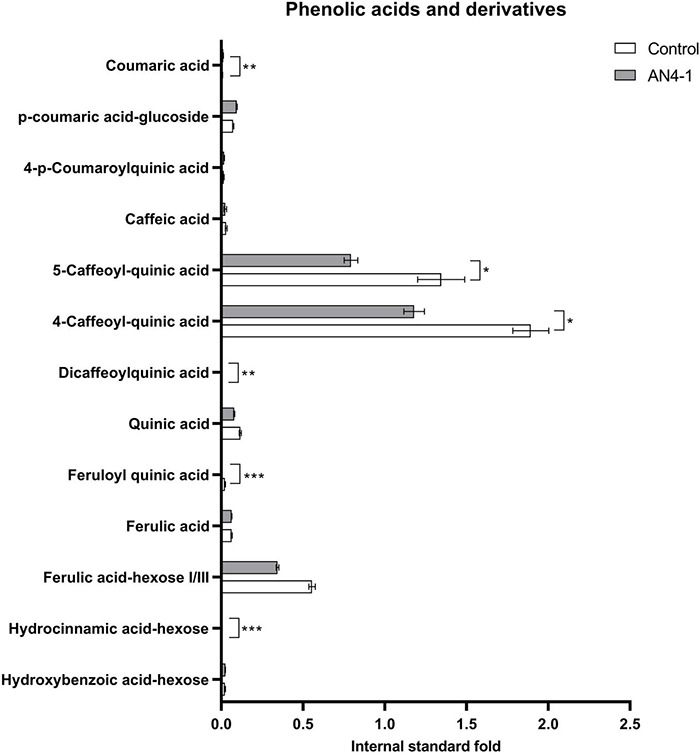
Graph representing phenolic compounds and their derivatives expressed as internal standard (formononetin) fold of control and AN4-1 HRCs. Columns report average values ± *SD* (*n* = 3). *p-value ≤ 0.05, **p-value ≤ 0.01 and ***p-value ≤ 0.001.

**FIGURE 4:**
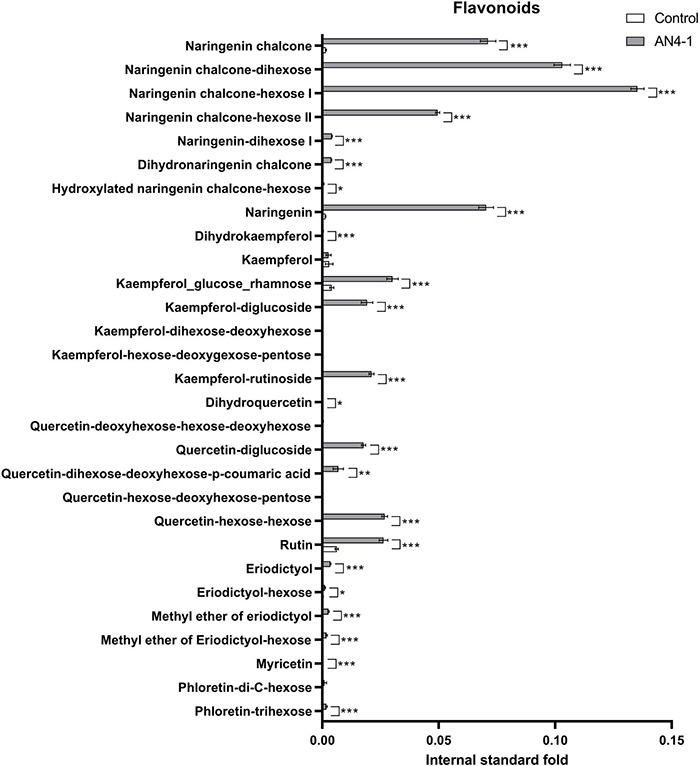
Flavonoids are expressed as internal standard (formononetin) fold of control and AN4-1 HRCs. Columns report average values ± *SD* (*n* = 3). *p-value ≤ 0.05, **p-value ≤ 0.01 and ***p-value ≤ 0.001.

To evaluate the effect of the accumulation of anthocyanins on their precursors and the final balance on flavonoids accumulation, a detailed analysis of phenylpropanoids was carried out by LC-HRMS on the engineered HRCs. A graphical representation of the accumulation levels of anthocyanins precursors is shown for the best anthocyanin-accumulating AN4-1 HRC (Figures 3, 4) and the other engineered HRC (Supplementary Files 1, 2). A series of phenolic acids and their derivatives (e.g., dicaffeoylquinic, 5-caffeoyl-quinic, and 4-caffeoyl-quinic acids) were accumulated at a significantly lower level compared to control. This finding might be ascribed to the role of these compounds as flavanones and flavonols precursors of their sugar-decorated derivatives and of anthocyanins that, in turn, resulted to be enhanced in accumulation in engineered HRCs. As a consequence of the lower accumulation of phenolic acids, hydroxycinnamic acid accumulated at reduced levels in engineered HRCs, with respect to control. Interestingly, the hydroxybenzoic acid level was not significantly different. Significantly higher levels of other valuable phenolics such as coumaric acid and caffeic acid derivatives were found in AN4-1 HRC compared to control (Figure 3).

Accordingly, among flavonoids, both flavanones (i.e., naringenin chalcone and its sugar-decorated derivatives, naringenin, eriodictyol) and flavonols (i.e., dihydrokaempferol, sugar-decorated kaempferol, dihydroquercetin, quercetin derivatives, rutin, and myricetin) resulted in an overall significantly higher accumulation in engineered HRCs compared to control (Figure 4 and Supplementary File 2). Notably, and coherently with phenolic acids precursor function, the observed fold change levels on most flavonoid groups (from naringenin and kaempferol derivatives to quercetin derivatives and rutin) displayed a much larger extent (192.82 ± 39.1 for naringenin dihexose I) compared to the phenolic acid precursor (0.623 ± 0.069 for 4-caffeoyl-quinic acids).

AN4-1 HRC was chosen as the candidate to perform the subsequent transcriptome analysis due to the higher content of the different anthocyanin and flavonoid species compared to the remaining clones.

### Transcriptome Analysis of Tomato *PhAN4* Hairy Roots

**FIGURE 5:**
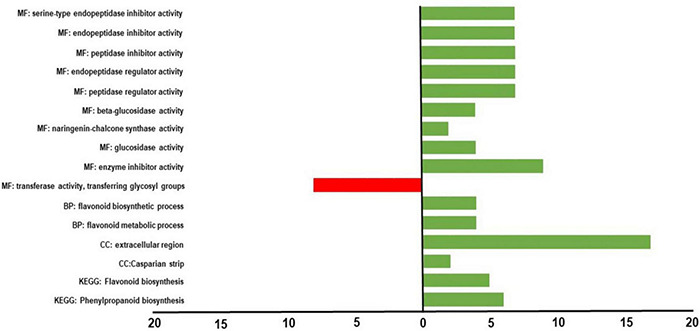
GOs distribution of differentially expressed genes (DEGs). Gene Ontology (GO) terms associated with upregulated (green bars) and downregulated (red bars) genes in the PhAN4 HRCs compared to control HRCs, based on “Molecular Functions” (MF), “Biological Process” (BP), “Cellular Component” (CC) and KEGG ontological domain.

The response to the constitutive expression of *PhAN4* in tomato HRCs were analyzed transcriptome-wide by RNAseq analysis and compared to control HRCs. AN4-1 showed a total of 442 differentially expressed genes (DEGs), of which 331 were upregulated and 111 were downregulated (Table 1 and Supplementary File 3). After Gene Ontology Enrichment (GOE) analysis, 38 upregulated and 8 downregulated genes were termed (Supplementary File 4). For upregulated DEGs, 3 GO categories were assigned: Molecular Function (MF), Biological Process (BP), and Cellular Component (CC) (Figure 5).

**TABLE 1:** List of the more representative and significant DEGs obtained from the GOE and functional annotation analyses in relation to agrospace application.

Gene ID	Log_2_Fc	Gene name	Function	References
Solyc03g020080.3.1	2.017930333	*SlPI11*	Pin-II type proteinase inhibitor/**biotic (herbivorous and insects resistance), abiotic (drought/heat)**	Fan et al., 2020
Solyc03g020030.3.1	10.72904412	*SlPI16*	Pin-II type proteinase inhibitor/**biotic (wound stress, insect resistance), abiotic (heat)**	Fan et al., 2020
Solyc11g020960.2.1	2.362943887	*SlPI51*	Proteinase inhibitor II/**biotic (wound stress)**	Fan et al., 2020
Solyc08g080630.3.1	2.33279266	*SlPI31*	Proteinase inhibitor 1/**biotic (TSWV infection), abiotic (drought)**	Fan et al., 2020
Solyc10g086090.2.1	2.247200225	*SlPI47*	Trypsin inhibitor 1/**abiotic (drought)**	Fan et al., 2020
Solyc10g086100.2.1	2.406338004	*SlPI48*	Proteinase inhibitor/**abiotic (heat)**	Fan et al., 2020
Solyc01g059965.1.1	3.476158885	*SlGluB*	Beta-1,3-glucanase/**biotic (defense against pathogens: *C. fulvum*, *P. infestans*)**	van Kan et al., 1992; Fan et al., 2021
Solyc09g091510.3.1	12.25514456	*CHS*	Chalcone synthase 1/**phenylpropanoids biosynthesis**	Zhang et al., 2019
Solyc05g053550.3.1	9.83030415	*CHS*	Chalcone synthase 2/**phenylpropanoids biosynthesis**	Kalt et al., 2020
Solyc02g083860.3.1	6.299607501	*F3H*	Flavonoid-3-hydroxylase/**phenylpropanoids biosynthesis**	Aoki et al., 2010; Zhang et al., 2019
Solyc02g085020.3.1	10.09460014	*DFR*	Dihydroflavonol reductase/**phenylpropanoids biosynthesis**	Bongue-Bartelsman et al., 1994
Solyc01g106650.3.1	3.003680242	–	Xyloglucan endotransglucosylase/**cell elongation**	Li P. et al., 2020
Solyc11g011210.2.1	2.189943987	*RSI-1*	RSI-1 precursor/**lateral root initiation**	Taylor and Scheuring, 1994
Solyc03g093390.3.1	2.390710296	*LeEXPB2*	Expansin-B15-like/**sexual reproduction**	Sundaresan et al., 2016
Solyc08g077910.3.1	3.227703527	–	Expansin-like B1/**sexual reproduction**	Nveawiah-Yoho et al., 2013
Solyc05g052245.1.1	2.186779111	–	Expansin A8-like/**cell wall organization**	–
Solyc09g010860.3.1	2.630126243	*EXPA4*	Expansin 4-like/**cell wall organization**	–
Solyc02g062510.3.1	2.392338587	–	Peroxidase 72-like/**phenylpropanoids biosynthesis**	Nveawiah-Yoho et al., 2013
Solyc04g080760.3.1	2.42913063	–	Peroxidase 9/**abiotic stress (hypoxia tolerance)**	Andolfo et al., 2014
Solyc02g077300.2.1	7.236518572	–	Peroxidase 19/**phenylpropanoids biosynthesis, biotic (ToMV infection)**	Andolfo et al., 2014; Safavi-Rizi et al., 2020
Solyc06g054320.1.1	2.690758759	–	Dirigent protein/**lignin biosynthesis**	Paniagua et al., 2017
Solyc10g055190.1.1	5.216357243	–	Dirigent protein/**lignin biosynthesis**	Paniagua et al., 2017
Solyc10g055200.1.1	2.06850204	–	Dirigent protein/**lignin biosynthesis**	Paniagua et al., 2017
Solyc04g010270.1.1	2.350516375	–	Dirigent protein/**lignin biosynthesis**	Paniagua et al., 2017
Solyc02g076710.3.1	2.307987898	*CathB*	Cathepsin B-like cysteine/**biotic (hypersensitive response)**	McLellan et al., 2009
Solyc05g053890.2.1	−2.032569214	–	UDP-GT-like/**flavonoids glycosylation**	Fernandez-Moreno et al., 2016
Solyc01g096560.2.1	4.109312617	*TomLOXD*	Subtilisin-like protease/**biotic (wound stress, resistance to insects and necrotrophic pathogens)**	Yan et al., 2013
Solyc07g054840.3.1	3.341226163	*AtMYB41*	Transcription factor 41/**abiotic (salt tolerance)**	Hoang et al., 2012
Solyc03g095810.3.1	6.923791313	–	Trichome birefringence-like/**xylan acetylation (resistance against micro-organisms, cold and drought)**	Zhang et al., 2020
Solyc10g007970.2.1	6.961895177	–	Transcription factor 77/**abiotic (water deficit)**	Asins et al., 2021
Solyc07g043690.2.1	2.800732902	*SlNPR1*	3-Hydroxyisobutyryl-CoA hydrolase/**abiotic (drought stress)**	Li J. et al., 2019
Solyc03g096460.3.1	2.302142017	–	**Wound signaling**	Scranton et al., 2013
Solyc02g080790.3.1	3.317661852	*SlDHS*	Deoxyhypusine synthase/osmotic stress and chilling injury	Wang et al., 2001; Gupta et al., 2013
Solyc10g081300.1.1	2.564715	*SlMC8*	Metacaspase 9/**biotic (apoptosis induction by pests), abiotic (drought, cold and salt)**	Liu et al., 2016
Solyc10g080690.2.1	2.304387313	–	Patatin/**abiotic (flooding)**	de Ollas et al., 2021
Solyc06g073760.3.1	4.071580028	*BGL2*	Beta-glucosidase/**sugar/organic acid ratio tomato fruits/resistance against different pathogens/softening**	Liu et al., 2016
Solyc12g035225.1.1	9.263765052	*RICESLEEPER1*	BED zinc-finger/**abiotic (salt tolerance)**	Kashyap et al., 2020
Solyc12g010500.2.1	5.358159809	–	E3 ubiquitin protein/**biotic (resistance to *X. perforans*)**	Shi and Panthee, 2020
Solyc12g010670.1.1	5.364030417	–	E3 ubiquitin protein/**biotic (resistance to *X. perforans*)**	Shi and Panthee, 2020
Solyc12g009630.2.1	5.183567126	*SlCaM3*	Calcium-binding protein/**biotic (*B. cinerea*), abiotic (mechanical wounding, salt and cold stress)**	Peng et al., 2014; Shi and Du, 2020
Solyc02g067750.3.1	6.915338794	*CA1*	Carbonic anhydrase/**abiotic (drought stress)**	Li X. et al., 2020
Solyc08g075705.1.1	4.258074963	*pTRX y2*	Thioredoxin Y2/**ROS detoxification, redox signaling network regulation**	Serrato et al., 2013
Solyc09g007190.3.1	−2.218315202	*PRXL2A*	Peroxiredoxin-like 2A/**redox regulatory protein**	–
Solyc03g098760.2.1	−5.109469505	–	I3 Kunitz-type trypsin inhibitor/**biotic (infection *Tetranychus* species infection)**	Islam et al., 2015; Schimmel et al., 2018
Solyc09g089490.3.1	−5.211378167	*SlPI40*	Proteinase inhibitor/**abiotic (drought, salt) biotic (*B. cinerea*, TSWV infection)**	Fan et al., 2020
Solyc06g008760.1.1	−3.056115855	–	Glutaredoxin-C13-like/**upregulated in tomato *rin* mutants**	Kumar et al., 2016
Solyc07g055610.2.1	−2.055605009	*PR1*	Resistance protein R1/**biotic (resistance to late blight and *F. oxysporum* infection)**	Bournival et al., 1989; Pan et al., 2000
Solyc01g010480.3.1	−2.123574853	*KAT1*	K+ channel KAT1/**abiotic (tolerance to potassium deficiency)**	Zhao et al., 2018
Solyc01g102610.3.1	−3.607916378	*FRO6*	Ferric reduction oxidase 6/**biotic (*P. solani* infection)**	Buoso et al., 2019
Solyc05g010320.3.1	4.308610241	*CHI*	Chalcone isomerase/**phenylpropanoids biosynthesis**	Morita et al., 2014; Li Z. et al., 2019
Solyc05g052240.3.1	8.452278685	*CHI*	Chalcone isomerase/**phenylpropanoids biosynthesis**	Ron et al., 2014; Hou et al., 2017
Solyc11g066580.2.1	16.35234434	*F3′5′H*	Flavonoid-3′,5′-hydroxylase/**phenylpropanoids biosynthesis**	Shi and Panthee, 2020
Solyc08g080040.3.1	8.861102345	*ANS*	Anthocyanin synthase/**phenylpropanoids biosynthesis**	Shi and Panthee, 2020
Solyc04g078140.3.1	8.35041607	*DilFl*	Cytochrome B5/**phenylpropanoids biosynthesis (essential for full activity of F3′5′H)**	de Vetten et al., 1999
Solyc09g082660.3.1	15.05027118	*OMT*	Caffeoyl-CoA-*O*-methyltransferase/**phenylpropanoids biosynthesis**	Roldan et al., 2014; Shi and Panthee, 2020
Solyc02g062975.1.1	7.081891304	*3UFGT*	UDP-glucose flavonoid 3-*O*-glucosyl transferases/**phenylpropanoids biosynthesis**	Hu et al., 2011; Tohge et al., 2020
Solyc10g083440.1.1	9.972475187	*3UFGT*	UDP-glucose flavonoid 3-*O*-glucosyl transferases/**phenylpropanoids biosynthesis**	Hu et al., 2011; Tohge et al., 2017
Solyc12g098590.2.1	16.55105848	*3UFGT*	UDP-glucose flavonoid 3-*O*-glucosyl transferases/**phenylpropanoids biosynthesis**	Hu et al., 2011; Tohge et al., 2020
Solyc09g059170.2.1	8.326262972	*3RT*	Anthocyanidin-3-*O*-glucoside rhamnosyltransferase/**phenylpropanoids biosynthesis**	Tohge et al., 2020
Solyc12g088170.2.1	13.21347105	*AAT*	Flavonoid-3-*O*-rutinoside-4”’-*O*-phenylacyltransferase/**phenylpropanoids biosynthesis**	Florio et al., 2021
Solyc03g025190.4.1	16.80814437	*MTP77*	Transparent testa 2-like/**toxic compound extrusion, regulation of cell turgescence**	dos Santos et al., 2017
Solyc10g006120.2.1	7.357873128	*LDOX*	Leucoanthocyanidin dioxygenase/**phenylpropanoids biosynthesis**	Pelletier et al., 1997
Solyc07g052490.3.1	7.173073383	*Atv*	Myb-like transcription factor Atv/**anthocyanin biosynthesis**	Cao et al., 2017
Solyc10g086290.2.1	8.651275639	*SlAN2*	AN2-like transcription factor/**anthocyanin biosynthesis**	Sun C. et al., 2019
Solyc12g005800.2.1	7.517786731	*SlMYBATV-like*	R3-MYB repressor/**anthocyanin biosynthesis**	Cao et al., 2017
Solyc09g065100.2.1	14.18672027	*AN1*	AN1-like transcription factor/**anthocyanin biosynthesis**	Qiu et al., 2016
Solyc10g084380.1.1	3.439413134	*PH3*	WRKY transcription factor/**anthocyanin biosynthesis**	Verweij et al., 2016
Solyc10g083900.2.1	3.542474125	*Myb27*	MYB transcription factor/**inhibitor of anthocyanin biosynthesis**	Albert et al., 2011, 2014
Solyc01g095640.2.1	4.928760848	*SlTRY*	Trichome initiation factor ECT3/**anthocyanin biosynthesis**	Tominaga-Wada et al., 2013; Kim et al., 2017
Solyc01g105880.4.1	3.469067651	*TPS4*	Monoterpenoid synthase/**terpene biosynthesis**	Velázquez-Márquez et al., 2021
Solyc09g092470.2.1	5.695783056	*TPS14*	Sesquiterpene synthase/**terpene biosynthesis, biotic (*F. oxysporum* resistance)**	Velázquez-Márquez et al., 2021
Solyc11g017240.2.1	3.78371117	*SlCM2*	Chorismate mutase/**volatile compounds biosynthesis, abiotic (drought stress)**	Tzin et al., 2015; Filiz et al., 2019
Solyc08g008630.3.1	−3.340796451	*Dwarf27*	Beta-carotene isomerase D27/**strigolactone and beta-carotene biosynthesis, biotic (psyllid resistance)**	Harrison et al., 2021
Solyc04g050930.3.1	−2.072953851	*VDE*	Violaxanthin de-epoxidase VDE/**carotenoid biosynthesis, biotic (*P. syringae* infection), abiotic (anoxia)**	Yang Y. X. et al., 2015
Solyc05g010180.3.1	−2.221215961	*CRTISO*	Carotenoid isomerase/**carotenoid biosynthesis**	Isaacson et al., 2002
Solyc12g006140.2.1	4.364619823	–	Chlorophyll a/b-binding protein/**fruits ripening**	Zouari et al., 2014
Solyc02g065220.3.1	−2.141082227	–	Cytochrome P450/**fruits ripening (extended shelf-life**	Gao et al., 2018
Solyc09g066150.1.1	−4.574546484	–	Cytochrome P450/**fruits ripening (extended shelf-life)**	Gao et al., 2018
Solyc07g006570.3.1	4.221848981	–	Ribonuclease 3-like/**RNA biogenesis**	Consortium et al., 2012
Solyc02g065230.3.1	3.170387722	*IMT7*	Cytochrome P450	–
Solyc02g091440.2.1	2.129583909	*bHLH83-like*	bHLH/**enhance root hair initiation, promote flowering in short day and maintain the iron balance**	Qian et al., 2021
Solyc12g088130.2.1	4.327213562	*bHLH93-like*	bHLH/**enhance root hair initiation, promote flowering in short day**	–
Solyc04g077780.3.1	2.019567752	–	LIM transcription factor/**cytoskeleton organization**	–
Solyc12g013850.2.1	−2.036803744	–	Glycosyltransferase/**regulates anther development and male-sterility**	Omidvar et al., 2015
Solyc02g081340.3.1	10.53476424	*GST*	Glutathione-*S*-transferase/**anthocyanin biosynthesis**	Alfenito et al., 1998
Solyc01g058030.2.1	8.08401203	–	Gibberellin 2-beta-dioxygenase/**determines dwarf phenotype with shorter internodes**	Sun X. et al., 2019
Solyc05g054360.3.1	6.837513657	–	Pectin methylesterase/**flower initiation**	Wen et al., 2020
Solyc12g010500.2.1	5.358159809	–	U-box protein/**biotic (resistance to *X. perforans***	Shi and Panthee, 2020

*The function was provided by SolGenomics Network annotation and by specific literature (in bold).*

#### Gene Ontology Enrichment Analysis

Molecular function (MF) resulted in the most abundantly represented GO category, with 9 GO terms and an intersection size of 15 genes over a query size of 165 genes. The most enriched terms were related to ‘enzyme inhibitory activity,’ with nine upregulated DEGs, seven of which are classified as serine-type endopeptidase inhibitors in tomatoes. Among them, Solyc03g020080.3.1, Solyc03g020030.3.1, Solyc11g020960.2.1, Solyc08g080630.3.1, Solyc10g086090.2.1, Solyc10g086100.2.1 encode specific Pin-II type proteinase inhibitors or protease inhibitors that have been already demonstrated to be players of the defense against wounding, pathogens, and pests and of response to abiotic stress, especially UV, drought, and heat stress in tomato (Bergey et al., 1996; Conconi et al., 1996; Fan et al., 2020). Within MF also ‘beta-glucosidase’ and ‘glucosidase activity’ terms were represented with four upregulated DEGs. Among them, Solyc01g059965.1.1 has been demonstrated to be implicated in the defense of tomato plants against pathogens (Murata et al., 2019), and its expression has been found to be modulated in tomatoes upon infection with *Cladosporium fulvum* and *Phytophthora infestans* (van Kan et al., 1992; Fan et al., 2021). Upregulation of Solyc06g073760.3.1 has been correlated to increased softening of transgenic tomato overexpressing a MADS-box transcription factor, affecting fruit development and ripening (Huang et al., 2017).

Within the BP category, coherently with secondary metabolites analysis, the ‘flavonoid biosynthetic process’ term was found. In particular, GOE analysis highlighted the upregulation of four genes: Solyc05g053550.3.1, Solyc09g091510.3.1, Solyc02g083860.3.1, Solyc02g085020.3.1. Solyc09g091510.3.1, and Solyc05g053550.3.1 are tomato *chalcone synthase* (*CHS*) I and II, respectively, that are early pathway genes that determine the accumulation of the naringenin chalcone precursor of anthocyanins and other flavonoids (Zhang et al., 2019). Solyc02g083860.3.1 is tomato *Flavonoid-3-hydroxylase* (*F3H*) that leads to the accumulation of dihydromyricetin, dihydrokaempferol, and dihydroquercetin precursors of anthocyanins and of other flavonoids in tomatoes (Aoki et al., 2010; Zhang et al., 2019). Solyc02g085020.3.1 is tomato *dihydroflavonol reductase* (*DFR*) that leads to the accumulation of leucoanthocyanidins in tomatoes (Bongue-Bartelsman et al., 1994).

Within the CC GO category, ‘Extracellular Region’ and ‘Casparian Strip’ terms were categorized. Seventeen genes over a query size of 115, were found to be upregulated within these terms. These genes, in many cases, are specifically correlated to lignin biosynthesis, cell wall organization, and resistance to abiotic and biotic stress functions in tomatoes. In particular, Solyc05g052245.1.1 and Solyc09g010860.3.1 are the tomato expansins A8-like and EXPA4, involved in cell wall organization (Consortium et al., 2012). Solyc02g062510.3.1, which shares 86% homology with the *S. tuberosum* Peroxidase 72-like gene, and Solyc02g077300.2.1 (tomato Peroxidase 19) are involved in the phenylpropanoids biosynthesis (Mei et al., 2009). In addition, also Solyc04g080760.3.1 (tomato Peroxidase 9) plays a role in hypoxia tolerance and maintaining the iron balance in tomato (Safavi-Rizi et al., 2020). Peroxidase 9 was also demonstrated to be a pathogenesis-related protein upregulated upon *Tomato Mosaic Virus* infection (Andolfo et al., 2014). Solyc06g054320.1.1, Solyc10g055190.1.1, Solyc10g055200.1.1, and Solyc04g010270.1.1 represent tomato dirigent proteins that contribute to the dimerization of conyferil alcohol, a crucial step toward lignin biosynthesis, modulating cell wall metabolism during abiotic and biotic stress exposure in tomato (Paniagua et al., 2017). Solyc01g110110.3.1 and Solyc02g076710.3.1 are tomato cysteine proteinases. In particular, Solyc02g076710.3.1 shares 90% homology with *N. benthamiana* cathepsin B-like cysteine proteases that are involved in the hypersensitive response (McLellan et al., 2009). Solyc08g080630.3.1, already highlighted as an upregulated DEG within the MF category GOE, is the tomato *SlPI31* protease inhibitor. This gene has been demonstrated to be upregulated under *Tomato Spotted Wilt Virus* infection in tomato roots and leaves and has been shown to be upregulated in drought-tolerant tomato lines and drought-sensitive varieties under drought conditions (Fan et al., 2020).

Gene ontology enrichment (GOE) analysis revealed only one downregulated DEG (Supplementary File 4) belonging to the Molecular Function term ‘glycosyl transferase.’ Solyc05g053890.2.1 represents the complete sequence of the tomato UDP-GT-like which is probably related to glycosylation of flavonoids prior to their transport to the vacuole (Solyc04g016200.1.1, Solyc04g016210.3.1, Solyc05g053890.2.1, Solyc01g095760.3.1 are incomplete sequences of tomato UDP-GT-like) (Fernandez-Moreno et al., 2016).

#### Differentially Expressed Genes Analysis

Despite no GOE being found in the Response to stimulus category, a relatively high number of DEGs was found that could be associated with response to abiotic and biotic stress response. Fifteen upregulated and seven downregulated DEGs were found to be correlated with such response. Among upregulated DEGs involved in early signals of defense responses against environmental cues, Solyc01g096560.2.1 (Log_2_Fc = 4,10; *TomLOXD*; Supplementary File 3), was retrieved. *TomLOXD* encodes a lipoxygenase that has been demonstrated to elevate wound-induced jasmonate response, upregulation of wound-induced genes, and enhanced resistance to insects and necrotrophic pathogens in tomatoes (Yan et al., 2013). Solyc07g054840.3.1 (Log_2_Fc = 3.34; tomato transcription factor 41) shares the best homology with Myb41 of *A. thaliana* where it functions as a Map-kinase involved in several signaling pathways that control plant development and salt stress tolerance (Hoang et al., 2012). Solyc03g095810.3.1 (Log_2_Fc = 6.92; tomato Trichome birefringence-like protein) upregulation mediates xylan acetylation and has been demonstrated essential in tomatoes for invading microorganism resistance and against environmental stress like cold and drought (Zhang et al., 2020). Solyc10g007970.2.1 (Log_2_Fc = 6.96; tomato WRKY transcription factor 77) has been demonstrated to be involved in the signaling to water deficit in tomatoes (Asins et al., 2021). Solyc07g043690.2.1 (Log_2_Fc = 2.80; tomato *SlNPR1*) has been suggested to regulate tomato plant drought response (Li J. et al., 2019). Solyc03g096460.3.1 (Log_2_Fc = 2.3) is a known modulator of wound signaling in tomatoes (Scranton et al., 2013). Solyc02g080790.3.1 (Log_2_Fc = 3.31; tomato deoxypusine synthase *SlDHS*) has been shown to be upregulated during osmotic stress and chilling injury (Wang et al., 2001; Gupta et al., 2013). Solyc10g081300.1.1 (Log_2_Fc = 2.56; *SlMC8*), encodes a metacaspase that is upregulated during apoptosis induction by pests and regulated by drought, cold, and salt in tomatoes (Liu et al., 2016). Solyc10g080690.2.1 (Log_2_Fc = 2.3; tomato patatin defense protein) has been demonstrated to be upregulated upon soil flooding in tomatoes (de Ollas et al., 2021). Solyc06g073760.3.1 (Log_2_Fc = 4.07; tomato β 1,3-glucanase 2 *BGL2*) has been found to be negatively correlated with the sugar/organic acid ratio of tomato fruits (Li et al., 2021). The simultaneous upregulation of *BGL2* and *PR1*, the marker genes of the salicylic acid (SA) pathway, is a hallmark of systemic resistance induced in tomato plants against different pathogens and can be followed by accumulation of SA at high levels (Peng et al., 2004; Fahim et al., 2016; Hanan et al., 2020). Solyc12g035225.1.1 (Log_2_Fc = 9.26; tomato putative zinc-finger domain-containing protein) shares 75% homology with rice *RICESLEEPER1* that was found to be upregulated in *S. chilense* in relation to transcription factors for salt tolerance (Kashyap et al., 2020). Solyc12g010500.2.1 and Solyc12g010670.1.1 (Log_2_Fc = 5.35 and 5.36, respectively) are categorized as E3 ubiquitin proteins that have been found to be upregulated in tomato genotypes resistant to *Xanthomonas perforans* (Shi and Panthee, 2020). Solyc12g009630.2.1 (Log_2_Fc = 5.18; tomato calmodulin *SlCaM3*), encodes an important calcium-binding protein that has been found to be upregulated in tomato stem and roots upon *Botrytis cinerea* infection and mechanical wounding (Peng et al., 2014). In addition, it has been reported that *SlCaM3* is strongly expressed under salt and cold stress in tomatoes (Shi and Du, 2020). Solyc02g067750.3.1 (Log*_2_*Fc = 6.91) shares 80% identity with *N. benthamiana CA1*. During drought stress CA1 proteins gradually diminish within the chloroplast and are accumulated in the cytosol, suggesting that they could be translocated from chloroplasts to the cytosol and act as a signal messenger from the chloroplast in tomato (Li P. et al., 2020). Importantly, in view of the possible improvement of resistance to abiotic stresses relevant to space, Solyc08g075705.1.1 (Log_2_Fc = 4.25; tomato plastidial thioredoxin Y2, *pTRX y2*) was found to be upregulated. PTRX y2 together with pTRX y1 and x-type TRXs is mostly involved in ROS detoxification and takes part in the complex redox signaling network regulating tomato plant development (Serrato et al., 2013).

Among downregulated DEGs correlated with resistance functions, Solyc09g007190.3.1 (Log*2*Fc = −2.21; tomato peroxiredoxin-like 2A PRXL2A) redox regulatory protein was found. Solyc03g098760.2.1 (Log_2_Fc = −5.1; tomato proteinase inhibitor I3 Kunitz-type trypsin inhibitor) has been demonstrated to be upregulated upon the cell-content feeding mite *Tetranychus* species infection (Schimmel et al., 2018) and it is told to protect seeds from predators (Islam et al., 2015). Solyc09g089490.3.1 (Log_2_Fc = −5.21; tomato SlPI40 protease inhibitor) can be induced by abiotic (drought and salt) and biotic (*Botrytis cinerea* and *Tomato Spotted Wilt Virus*) stress (Fan et al., 2020). Solyc06g008760.1.1 (Log_2_Fc = −3.05; tomato *Glutaredoxin-C13-like*) has been demonstrated to be upregulated in ripening inhibitor (*rin*) mutants of *S. lycopersicum* (Kumar et al., 2016). Solyc07g055610.2.1 (Log_2_Fc = −2.05); tomato Resistance protein R1) has been related to resistance to late blight (Bournival et al., 1989) and it has been shown to be linked to proteinase inhibitor I3 upon *Fusarium oxysporum* infection (Bournival et al., 1989; Pan et al., 2000). Solyc01g010480 (Log_2_Fc = −2.2; tomato K+ channel KAT1) was demonstrated to be highly upregulated in low K tolerant tomato genotypes upon potassium deficiency (Zhao et al., 2018). Solyc01g102610.3.1 (Log2Fc = −3.60; tomato FRO6) is involved in nutrient transport in phloem and was observed to be downregulated under *Phytoplasma solani* infection of tomato (Buoso et al., 2019).

**FIGURE 6:**
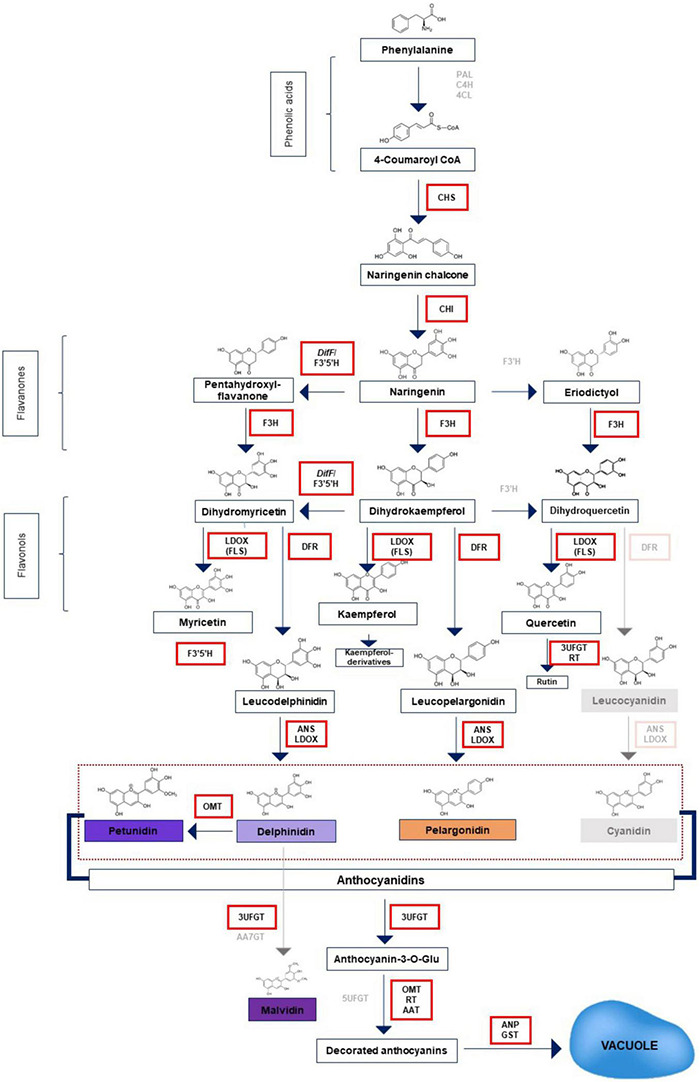
Schematic diagram of the anthocyanin biosynthetic pathway. The structural genes upregulated by ectopic expression of *PhAN4* in MicroTom HRCs and leading to accumulation of compounds are in black and marked by red boxes. PAL, phenylalanine ammonia-lyase; C4H, cinnamate-4-hydroxylase; 4CL, 4-coumarate CoA ligase; CHS, chalcone synthase; CHI, chalcone isomerase; F3′H, flavonoid-3′-hydroxylase; F3′5′H, flavonoid-3′,5′-hydroxylase; DFR, dihydroflavonol reductase; ANS, anthocyanin synthase; OMT, O-methyltransferase; AAT, anthocyanin acyltransferase; RT, rhamnosyltransferase; UFGT, UDP-glucose flavonoid 3-*O*-glucosyl transferase; AA7GT, cyanidin-3-*O*-glucoside-7-*O*-glucosyltransferase; ANP, anthocyanin permease; GST, glutathione-*S*-transferase.

Despite GOE retrieving only four genes among the ‘flavonoid biosynthetic process’ term, many other upregulated DEGs resulted in the analysis that is associated with biosynthesis of anthocyanins and other specialized metabolites (Figure 6). Twelve additional structural genes and six transcription factors were identified as upregulated DEGs. In addition to *CHS, F3H* and *DFR*, DEGs analysis revealed also tomato naringenin-chalcone isomerase (*CHI*) (Solyc05g010320.3.1; Log_2_Fc = 4.30 and Solyc05g052240.3.1; Log_2_Fc = 8.45), tomato flavonoid-3′,5′-hydroxylase *F3*′*5*′*H* (Solyc11g066580.2.1; Log_2_Fc = 16.35), tomato anthocyanin synthase *ANS* (Solyc08g080040.3.1, Log_2_Fc = 8.86) were found. Interestingly, also Solyc04g078140.3.1, which shares the best homology with *Cytochrome b5* of *P. hybrida*, where it is essential for full activity of *F3′5′H*, was upregulated (Log_2_Fc = 8.35) (de Vetten et al., 1999). In addition, tomato caffeoyl-CoA-*O*-methyltransferase Solyc09g082660.3.1; Log_2_Fc = 15.0503), already identified as the prime candidate gene responsible for anthocyanin methylation in tomatoes due to significant correlation of expression with *ANS, DFR*, and *F3′5′H* in *Rosea1* and *Delila* fruits (Roldan et al., 2014), resulted upregulated. It shares the best homology with *P. hybrida O*-methyltransferase (*OMT*). Interestingly, it was found to have a significant correlation with abiotic stress in tomatoes (Shi and Panthee, 2020). Three tomato UDP-glucose flavonoid 3-*O*-glucosyl transferases (*3UFGT*) (Solyc02g062975.1.1, Log_2_Fc = 7.08; Solyc10g083440.1.1, Log_2_Fc = 9.97; Solyc12g098590.2.1, Log_2_Fc = 16.55) catalyzing the transfer of the glucosyl moiety from UDP-glucose to the 3-hydroxyl group of anthocyanidins in tomato were found among upregulated DEGs, as well (Hu et al., 2011; Tohge et al., 2020). Furthermore, anthocyanidin-3-*O*-glucoside rhamnosyltransferase (*3RT*) (Solyc09g059170.2.1, Log_2_Fc = 8.3262), which in tomato controls the conversion of anthocyanidin-3-glucosides to anthocyanidin-3-rutinosides by the UDP rhamnose, was found among upregulated DEGs (Tohge et al., 2020). Tomato anthocyanin acyltransferase (*AAT*, or Flavonoid-3-*O*-rutinoside-4”’-*O*-phenylacyltransferase; Solyc12g088170.2.1, Log_2_Fc = 13.21) resulted upregulated, as well. Solyc02g081340.3.1 (Log_2_Fc = 10.53) resulted among upregulated DEGs. In tomato, it encodes a putative Glutathione S-Transferase that shares 84% homology with *P. hybrida GST* that is responsible for anthocyanin sequestration in the vacuole (Alfenito et al., 1998). Solyc03g025190.4.1 (Log_2_Fc = 16.80; tomato *MTP77*) resulted highly upregulated among DEGs. This gene belongs to clade 1 of the multidrug and toxic compound extrusion (MATE) family member (*Transparent testa 2-like*), which in tomato has been associated with vacuolar chloride channels related to the regulation of cell turgescence. In Micro-Tom, many MATE belonging to clade 1 have been functionally related to the transport of secondary metabolites (dos Santos et al., 2017). Solyc10g006120.2.1 (Log_2_Fc = 7) was found to be upregulated, as well. This gene shares 59% sequence identity with leucoanthocyanidin dioxygenase (*LDOX*) from *P. hybrida* and 61% with *S. tuberosum FLS* (Pelletier et al., 1997). LDOX has been demonstrated to be a bi-functional enzyme being able both to convert leucoanthocyanidins into anthocyanidins and to catalyze the *in planta* formation of flavonols in fls1-2 mutants of *A. thaliana* (Preuß et al., 2009).

Together with the regulation of structural genes, *PhAN4* positively modulated also transcription factors involved in anthocyanin biosynthesis. Solyc07g052490.3.1 (Log_2_Fc = 7.17, tomato Myb-like transcription factor *Atv*) is involved in anthocyanin biosynthesis (Cao et al., 2017). Also, Solyc10g086290.2.1 (Log_2_Fc = 8.65), which shares 83% homology with *Solanum tuberosum AN2-like*, is a R2R3Myb involved in anthocyanin biosynthesis, was found (Sun C. et al., 2019; Colanero et al., 2020; Yan et al., 2020). Solyc09g065100.2.1 (Log_2_Fc = 14.18) maps the Hoffman’s anthocyaninless (AH) locus and encodes a bHLH factor (SlAN1) that positively regulates anthocyanin biosynthesis in tomatoes (Qiu et al., 2016). No effects of *PhAN4* expression were found on another known regulator of anthocyanins biosynthesis, *AN11*. Additionally, Solyc10g084380.1.1 was upregulated (Log_2_Fc = 3.43). In *Solanum lycopersicum*, this gene encodes a WRKY that shares 76% homology with *PH3* from *Petunia hybrida* where it regulates vacuolar acidification and boosts anthocyanin biosynthesis, as well (Verweij et al., 2016).

Among upregulated DEGs, putative repressors of anthocyanins biosynthesis were found. The upregulated Solyc12g005800.2.1 (Log_2_Fc = 7.51) is the tomato SlMYBATV-like (Cao et al., 2017), a R3-MYB repressor. Solyc10g083900.2.1 (Log_2_Fc = 3.54; tomato R2R3Myb transcription factor 27) shares the best homology with *Myb27* from *P. hybrida*, where it is a repressor of the synthesis of anthocyanins (Albert et al., 2011, 2014) and Solyc01g095640.2.1 (Log_2_Fc = 4.92; tomato *ETC3* or *SlTRY*) orthologous in *A. thaliana* acts a repressor of anthocyanins accumulation, as well (Tominaga-Wada et al., 2013).

To complete the description related to the modulation of genes related to specialized metabolites, three DEGs resulted to be upregulated and three downregulated. Among upregulated DEGS, Solyc01g105880.4.1 and Solyc09g092470.2.1 (Log_2_Fc = 3.46 and 5.69, respectively; tomato monoterpenoid synthases 2 *TPS4* and *TPS14*), are involved in the biosynthesis of monoterpenes and sesquiterpenes, respectively. *TPS4* has been found to be upregulated in tomato cultivars resistant to *Fusarium oxysporum* (Velázquez-Márquez et al., 2021). Solyc11g017240.2.1 (Log_2_Fc = 3.78; tomato chorismate mutase 2 *SlCM2*), catalyzes the first step of the shikimate pathway from phenylalanine to the volatile compounds responsible for tomato fruit aroma and quality and defense from biotic and abiotic stress response (Tzin et al., 2015). In tomatoes, *SlCM2* has been also shown to be upregulated under drought stress (Filiz et al., 2019). Among downregulated genes, Solyc08g008630.3.1 (Log_2_Fc = −3.34; tomato chloroplastic beta-carotene isomerase D27 *Dwarf27*) is involved in strigolactone and beta-carotene biosynthesis and has been found to be downregulated in psyllid-infested tomato plants (Harrison et al., 2021). Solyc04g050930.3.1 (Log_2_Fc = −2.07; tomato violaxanthin de-epoxidase *VDE*) is involved in the carotenoid biosynthesis. Suppression of *VDE* can induce the photo-inhibition of the PSII and, at the same time, it results in an accumulation of fucoxanthin that functions as an efficient anti-oxidant in anoxia conditions (Tohge et al., 2020). Moreover, it has been shown that *VDE* is downregulated in tomato plants upon *Pseudomonas syringae* pv. tomato DC3000 infection (Yang Y. X. et al., 2015). Solyc05g010180.3.1 (Log_2_Fc = −2.22; tomato carotenoid isomerase *CRTISO*) is involved in carotenoids biosynthesis. In MicroTom fruits, the downregulation of carotenoid isomerase has been demonstrated to induce an accumulation of zeta-carotene and *cis*-prolycopene (dos Santos et al., 2017; Florio et al., 2021), both elevating and modifying carotenoid profiles toward more bioavailable forms compared to wild-type (Pelletier et al., 1997).

To complete the description related to DEGs that were modulated upon expression of *PhAN4* and that are related to plant physiology, in addition to Solyc06g073760.3.1 (already described in GOE analysis in the MF category), Solyc12g006140.2.1 (Log_2_Fc = 4.36; tomato chloroplastic light-harvesting chlorophyll a/b-binding protein 37) was found to be upregulated. Its upregulation was demonstrated to improve photosynthesis and in extending the shelf life in tomato plants (Zouari et al., 2014). Among downregulated DEGs, Solyc02g065220.3.1 (Log_2_Fc = −2.14; tomato cytochrome P450) was shown to have significantly different transcript levels between purple and red sectors of VIGs *Del/Ros* tomatoes late ripening. Its downregulation has been associated with extended shelf life (Gao et al., 2018). Solyc09g066150.1.1 (Log_2_Fc = −4.57; putative tomato cytochrome P450) suppression has been found in transgenic tomato plants unable to perform DNA methylation and its upregulation has been associated with fruit ripening (Zhao et al., 2018).

### Phenolic Content and Antioxidant (2,2-Diphenyl-1-Picrylhydrazyl) Activity

**FIGURE 7:**
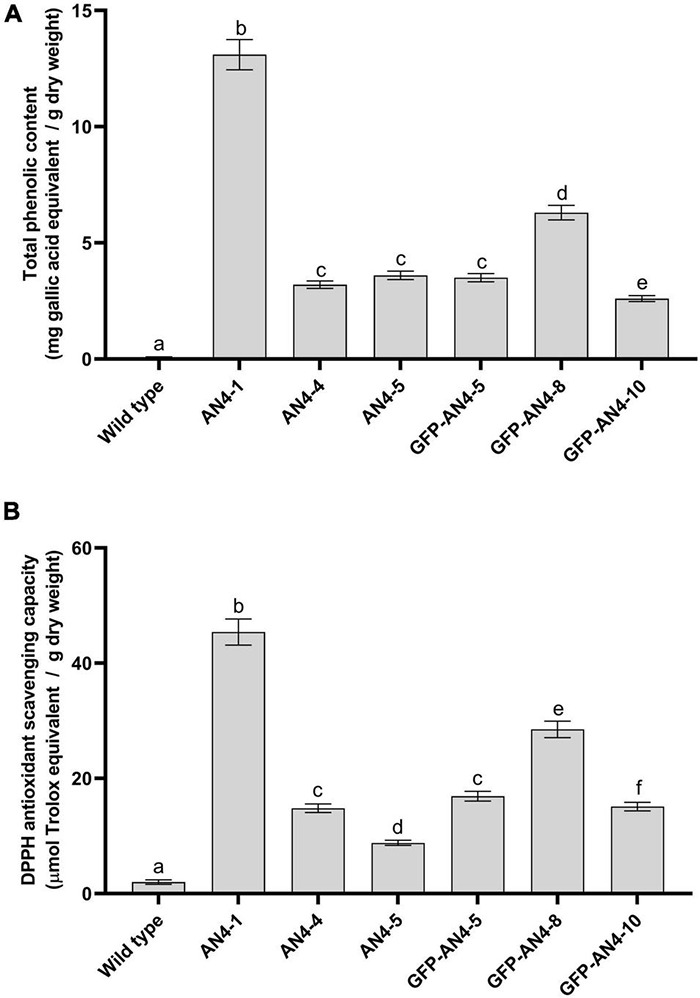
Total phenolic content in MicroTom transgenic HRCs reported in mg of gallic acid equivalents/g of dry weight **(A)**. Analysis of antioxidant activity (DPPH) in MicroTom transgenic HRCs reported as μg Trolox equivalents/g dry weight (DW) **(B)**. Each analysis consisted of triplicate measurements of each sample and data were averaged over the three measurements. A univariate statistical analysis based on Student’s *t*-test and one way-ANOVA (*P* ≤ 0.05) was carried out.

To verify whether *PhAN4* expression was associated with increased antioxidant activity, *in vitro* scavenging activity of the hydroalcoholic soluble fraction of HRCs was assessed by non-enzymatic 2,2-diphenyl-1-picrylhydrazyl (DPPH) assay (de Torre et al., 2019). The total phenolic content (expressed as μg of gallic acid equivalents (GAE)/g of dry weight, DW) was found to be ninefold and eightfold compared to control (18.2 × 103 μg GAE/g DW in AN4-1 and 16.4 × 103 μg GAE/g DW AN4-GFP-8 HRCs (Figure 7A). The trolox equivalent antioxidant capacity of the hydroalcoholic soluble fraction (containing anthocyanins) in purple HRCs appears to be thirty (in the AN4-1 clone) and twenty (in the AN4-GFP-8 clone) times higher than in control HRCs (Figure 7B).

### Electron Spin Resonance Analysis

**FIGURE 8:**
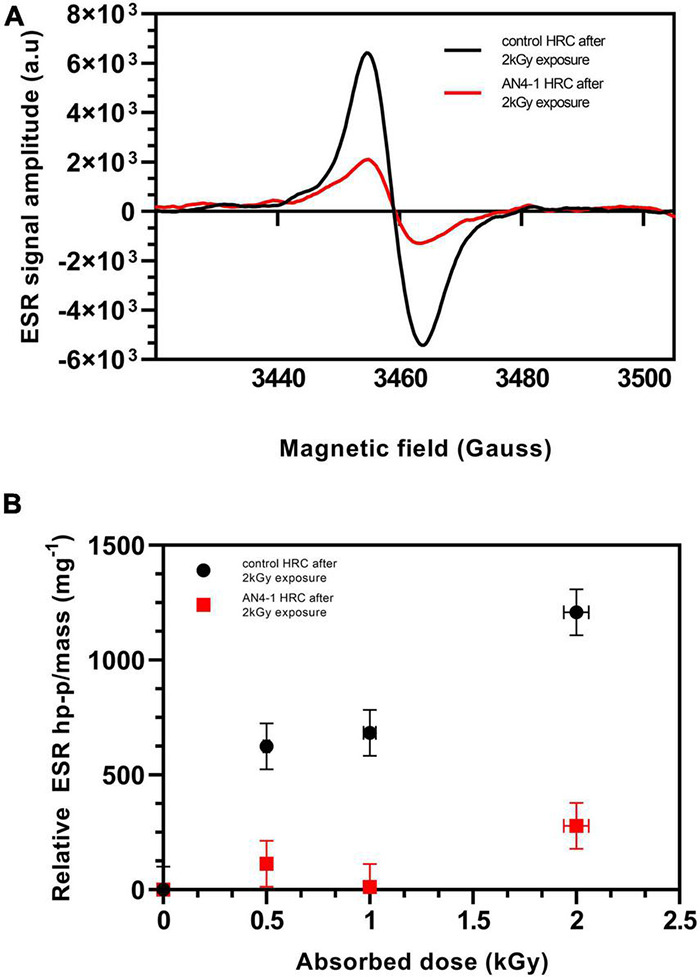
ESR spectra of AN4-1 and control HRCs gamma-irradiated at 2 kGy absorbed dose (dose rate = 1.8) **(A)**. ESR kGy water/h signal intensity of white and purple HRCs gamma are reported as a function of the absorbed dose (up to 2 kGy absorbed dose; dose rate = 1.8 kGy water/h) **(B)**. The intensity of each signal is expressed as the peak-to-peak height normalized for mass units and by subtracting the intensities of the HRCs signals before irradiation.

To further characterize the effect of the ectopic expression of *PhAN4*, MicroTom HRC lyophilized material was investigated for its ability to counteract the generation of reactive oxygen species (ROS) and for the maintenance of this feature after high dose gamma radiation. The AN4-1 HRC was chosen as the best candidate to evaluate resistance to radiation by Electron Spin Resonance (ESR). This analysis assessed the amount of peroxyl radicals in lyophilized HRCs. The HRCs ESR signal is characterized by a singlet signal at 3454 Gauss, correspondent to peroxyl radicals induced by gamma irradiation (Andersen et al., 2000; Ichikawa et al., 2001; Nagata et al., 2003; Rossetto et al., 2007; Esatbeyoglu et al., 2014; Faure et al., 2014). Negligible satellite peaks below 3,440 and above 3,480 Gauss are present as well and are related to several different contributions (such as cellulose-like molecules and anthocyanins) (Andersen et al., 2000; Tuner and Korkmaz, 2007; Petrisor et al., 2008; Esatbeyoglu et al., 2014; Faure et al., 2014). Typical ESR spectra of HRCs irradiated at 2,000 Gy absorbed dose, are shown in Figure 8A.

The AN4-1 and control spectra before gamma radiation exposure do not show significant peaks (data not shown). After irradiation (0.5, 1, and 2 kGy), the control shows a signal intensity significantly higher than AN4-1 (Figures 8A,B). In particular, AN4-1 shows only a negligible signal increase after irradiation from the lowest to the highest absorbed dose. On the contrary, control HRC shows a constant and nearly linear increase of singlet intensity, already evident at low absorbed doses.

### Ultraviolet-Visible Absorbance Spectra

**FIGURE 9:**
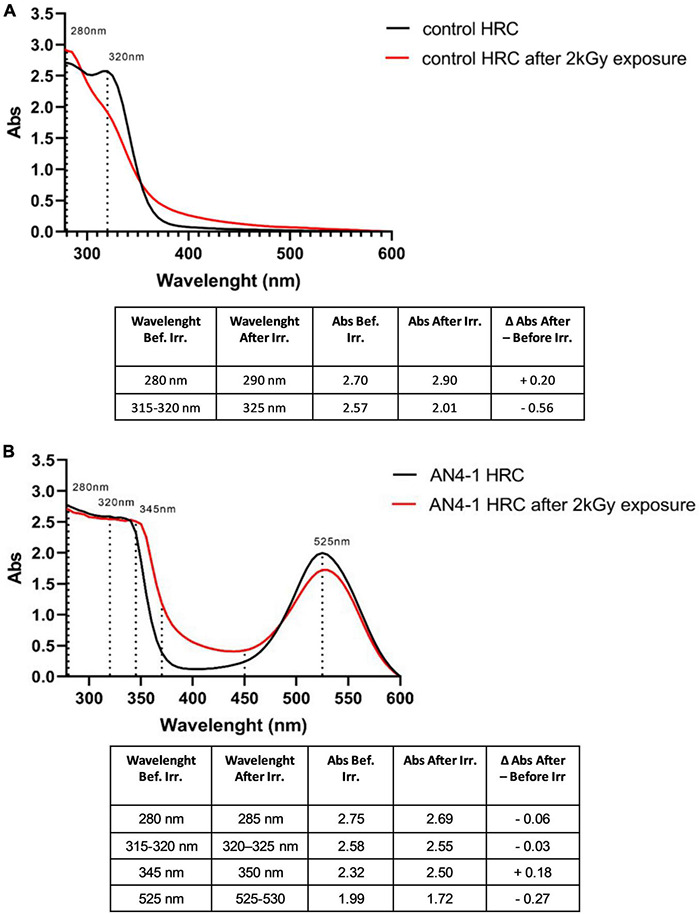
UV-VIS spectra of MicroTom HRCs before and after 2 kGy absorbed dose. In control, peaks at 280 nm and 320 nm indicate the presence of flavonols, hydroxycinnamic acids, tannins, and flavanols **(A)**. In AN4-1, the additional peak at 345 nm completes the flavonols group profile compared to control, while the peak at 525 nm represents anthocyanins. The curve in the region between 400 and 450 nm refers to possible glycosylation, precipitation, complexation of anthocyanins **(B)**.

We have used UV-VIS analysis of HRC extracts to characterize the resistance to gamma irradiation of the phenolic compounds accumulated in the AN4-1 line. Before irradiation, both control and AN4-1 acidic extracts revealed the main peak at 280 nm, followed by another peak at 315–320 nm (Figure 9).

In accordance with metabolomic data, in the spectra obtained in absence of irradiation, peaks around 280 nm and 320 nm indicate the presence of flavonols, hydroxycinnamic acids, tannins, and flavanols (Solìs-Oviedo and de La Cruz Pech-Canul, 2019). The shoulder at 345 nm together with that at 280 nm further defines the UV-VIS spectra of the flavonols in AN4-1 (Saha et al., 2021). Anthocyanins and anthocyanins associated with phenolic acids also produce peaks around 280 nm and 320 nm, respectively (Solìs-Oviedo and de La Cruz Pech-Canul, 2019), contributing to the profile of AN4-1 HRCs in Figure 7B. Anthocyanins result in an additional characteristic peak at 525 nm (Vivar-Quintana et al., 2002; da Silva et al., 2007; Fedenko et al., 2017), which, as expected, is observed in AN4-1 and not in control HRCs. Gamma irradiation determined a slight absorbance increase at 280 nm and a significant decrease at 320 nm in controls. On the contrary, these peaks remained unchanged in AN4-1. In AN4-1, the absorbance at 400–450 nm is possibly an indication of glycosylation, precipitation, complexation with tannins of anthocyanins upon irradiation (Saha et al., 2021). Gamma irradiation determined a slight decrease of the absorbance at 525 nm in AN4-1, as confirmed by a slight discoloration of the root material after irradiation.

### Photoluminescence Analysis

**FIGURE 10:**
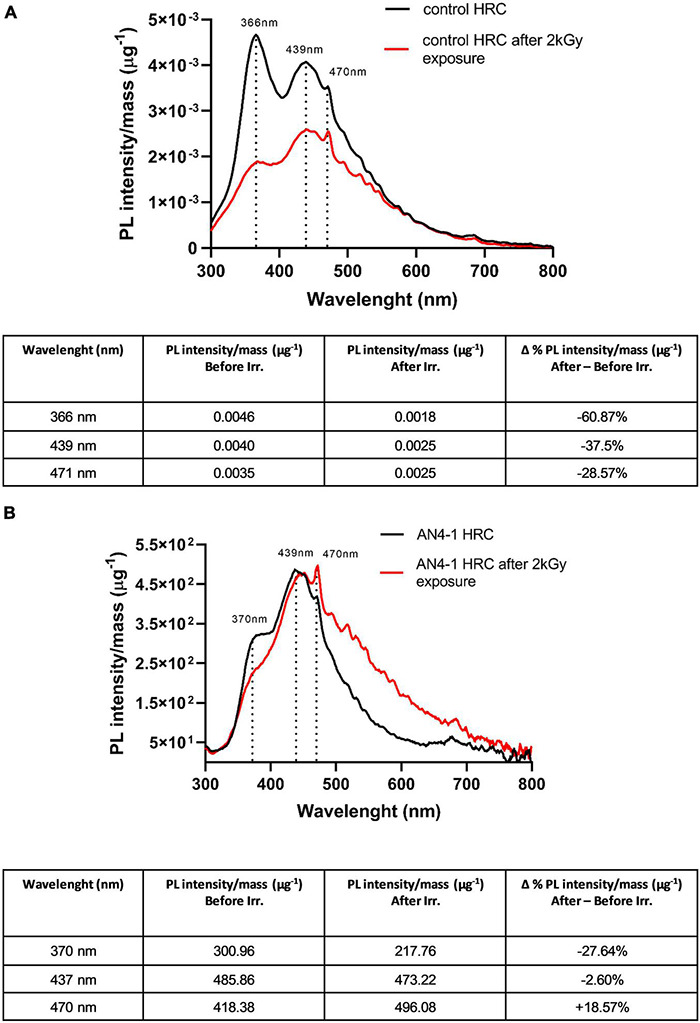
Photoluminescence spectra of control **(A)** and AN4-1 **(B)** HRCs and related mass-normalized emission intensities (tables) before and after 2 kGy gamma irradiation. Main peaks refer to tryptophan (370 nm), its degradation products kynurenine and 3-hydroxykynurenine (439 and 470 nm), and carbamate anion (470 nm).

Photoluminescence emission spectra were analyzed to determine the resistance to misfolding and oxidation of MicroTom HRCs soluble proteins after gamma irradiation. Photoluminescence spectra of extracts containing soluble proteins from control (Figure 10A) and AN4-1 HRCs (Figure 10B) were produced. The mass-normalized emission spectra of not irradiated soluble protein samples are similar in controls and AN4-1 and are characterized by high peaks mainly corresponding to tryptophan (370 nm) (Yang H. et al., 2015; Hilaire et al., 2017) and its metabolic products such as kynurenine (470 nm) and 3-hydroxykynurenine (439 nm) (Lohmann et al., 1988; Daly et al., 2009; Gakamsky et al., 2017). In addition, a peak at 470 nm is indicative of the presence of carbamate anions (Pan et al., 2013). In control HRC, the 2 kGy absorbed dose caused a decrease of fluorescence emission intensity for all three peaks. In particular, the Δ% photoluminescence intensity after and before the irradiation shows that the peak at 370 nm is reduced by 67.87%, the peaks at 439 nm and 470 nm by 37.5%, and 28.57%, respectively (Figure 8A). In AN4-1 only the 370 nm peak decreased (−27.64%), while a slight increase (+18.57%) of the kynurenine peak (470 nm) was present. No significant modifications were observed at 439 nm (Figure 10B).

## Discussion

Anthocyanins are well known for their antioxidant and health-protection properties. Tomato has been already subjected to genetic manipulation for improved levels and composition of these high-value compounds (Schauer et al., 2006; Klee and Tieman, 2013; Giovannoni, 2018; Wang et al., 2019). Nutraceutical improvement of tomato is expected to provide a nourishing food source for future long-term manned space missions such as NASA’s “Artemis” lunar exploration program. The Artemis initiative includes sending a suite of new technology demonstrators to establish a sustained human presence by 2028 (DeZwart and Henderson, 2021). Low-power systems to grow plants will have the role to provide fresh and nutritious food to supplement astronauts’ diet and provide psychological benefits. The experiments conducted in the VEGGIE module onboard the International Space Station over the last few years have pioneered this scenario (Wolff et al., 2014; Fu et al., 2016; Massa et al., 2016; Zabel et al., 2016; Imhof et al., 2018).

The space environment, totally unusual for plants, can affect their metabolic processes and, consequently, growth, due to high-energy ionizing radiation, microgravity, magnetic field, and ultra-vacuum (Williams et al., 2004; Lotito and Frei, 2006; Moghaddam et al., 2011; Thamaphat et al., 2015; van Hoeck et al., 2017). Among abiotic stresses that plants will have to cope with in extraterrestrial environments artificially adapted to space agriculture, pro-oxidant stimuli are, indeed, the most prominent, due to cosmic ionizing radiation. Acting directly and indirectly to delay oxidative damage, enzymatic players, and metabolites activating endogenous antioxidant defense systems may help plant growth in the space environment. ROS attack plant cells causing loss of their structure and function (Vandenhove et al., 2010; Moghaddam et al., 2011; Marcu et al., 2013a; Vardhan and Shukla, 2017; Gudkov et al., 2019), rapidly reacting with almost all structural and functional organic molecules in the plant cells and causing irreversible oxidative damage on DNA, lipids, and proteins (Scandalios, 2002; Sewelam et al., 2016). The issue of counteracting overproduction of free radicals generated by harmful ionizing radiation is crucial for human survival in space outposts, as well, and opens the way to the ideal ‘anti-oxidant space fresh food.’ Indeed, the oral intake of clinically tested chemical radioprotectants (i.e., thiols, aminothiols, thiadiazoles, and benzothiazoles) is limited due to toxicity (Copp et al., 2013). Therefore, there is great interest in the development of natural antioxidants possibly produced by plants and introduced with the diet (Gómez et al., 2021). Together with the above-mentioned abiotic ‘macro-stresses,’ plants intended for space agriculture will have to cope with a plethora of abiotic ‘micro-stresses’ related to the different cultivation environment/systems that will be adopted. Furthermore, plants will have to share with humans future crewed space habitats (spaceflights, planetary outposts, and life-support systems) where stringent microbial quality requirements may not be achieved (Amalfitano et al., 2020).

Plants have an intrinsic potential for adaptation as the heritage of ancestors that survived in the harsh initial terrestrial environments (de Vries and Archibald, 2018). The ‘design’ of plant ideotypes suitable to these environments may be achieved by further refinement of existing biochemical and physiological features, such as specialized metabolites, that plants use to survive stress. Novel genotypes boosting biosynthetic pathways for the production of specialized metabolites can be generated by manipulation of their regulators. Among regulators, there are MYB transcription factors, known for their contribution to the increased complexity of land plants and to be regulators of plant responses to the environment (Lloyd et al., 2017; Gao et al., 2018; Kashyap et al., 2020; Shi and Panthee, 2020). Members of this large family of regulators are key players in the modulation, among others, of the biosynthesis of flavonoids, like anthocyanins, in different plant parts and response to all kinds of stimuli (Ambawat et al., 2013; Liu et al., 2015; Roy, 2016). MicroTom is extremely compact, early yielding, and would be suitable for agriculture in the confined area of a crewed space module. Here, we described the ectopic expression of the anthocyanin R2R3-MYB regulator *PhAN4* from *Petunia hybrida* in MicroTom hairy roots as a testbed for future engineered whole plants. As known, anthocyanin production is regulated by R2R3-MYB, bHLH, WDR, and WRKY cooperation (MBWW transcriptional complex) (Spelt et al., 2000; Koes et al., 2005). In this complex, the R2R3-MYB transcription factor is probably the limiting factor, in that the WDR and bHLH proteins are thought to be constitutively expressed (Zhang et al., 2013, 2014). When specific MYB proteins are expressed in plants, this induces anthocyanin accumulation. Since some specific MYB transcription factors can function in regulating not only the biosynthesis of anthocyanins but also other traits (Meng et al., 2015; Vardhan and Shukla, 2017; Jian et al., 2019), we investigated if *PhAN4* may affect, together with accumulation of anthocyanins, features associated with improved traits related to survival in harsh and confined environments such as future space outposts.

Differently from the wealth of literature available on whole transgenic plants and cell culture systems, data concerning anthocyanins production are limited for hairy roots. To date, no data are available on anthocyanin accumulation in tomato hairy roots by metabolic engineering to make comparisons with our work. Although not comparable to levels achieved in transgenic plants (Butelli et al., 2008; Povero et al., 2011; Kiferle et al., 2015), accumulation of anthocyanins occurred in MicroTom HRCs upon *PhAN4* transformation, as expected due to the nature of the transgene. Importantly, no significant variation of transformed HRCs growth rates was found compared to controls. This gives a clue about the non-detrimental activity of *PhAN4* toward primary metabolism in this system.

In accordance with literature related to transgenic tomato plants (Butelli et al., 2008; Su et al., 2016; Jian et al., 2019), mass spectrometry allowed us to identify glycosylated and acylated delphinidin and petunidin as the most abundant anthocyanins in *PhAN4* HRCs. Interestingly, two additional anthocyanins, unusual in tomato, pelargonidin-3-glucoside, and malvidin-3-*O*-(4”’coumaroyl)-rutinose-5-*O*-glucose, were detected, as well. Malvidin and Pelargonidin derivatives have already been detected in *Del* and *Ros1* and *LC* and *C1* tomato plants, respectively (Bovy et al., 2002; Su et al., 2016). Interestingly, pelargonidin plays a major role in reducing genotoxic stress induced by environmental toxicants in plants (Khandelwal and Abraham, 2014). In addition, rather than other anthocyanins, pelargonidin-3-glucoside, that was accumulated in engineered MicroTom HRCs, has high bioavailability, being absorbed in an intact form into the gastrointestinal wall, undergoing first-pass metabolism and entering the systemic circulation as 4-hydroxybenzoic acid, a stable metabolite that is considered one of the main players of the systemic health effects of anthocyanins (Fang, 2014). In order to achieve a better understanding of *PhAN4* in the tomato hairy root phenylpropanoid pathway, we measured 14 phenolic acids and derivatives and 29 flavonoids: notably, a reduction in the content of some members of the former, and a massive increase in most of the compounds in the latter were observed, thus proving *PhAN4* is able to trigger the metabolic flux at both flavonoid and anthocyanin levels.

Data on the accumulation of anthocyanins and phenylpropanoids obtained by mass spectrometry was supported by transcriptome-wide RNA-seq analysis. RNA-seq, in complex, depicted a reprogramming oriented not only to anthocyanin accumulation, as expected, but also to positive regulation of cell response to biotic and abiotic stress and, possibly, to fruit quality-related traits.

Expression of *PhAN4* increased the transcript levels of almost all of the genes encoding enzymes required for anthocyanin biosynthesis with the exception of *PAL, C4H, 4CL, F3′H*, and *5UFGT*. To facilitate comparisons, transcriptomic research on tomato plants for anthocyanins enrichment has been summarized in Table 2. *CHS* and *CHI* had been already found to be upregulated by overexpression of *SlAN2* in tomatoes, but not by ectopic expression of *Del/Ros* that, in turn, were able to upregulate *F3H* (Butelli et al., 2008; Jian et al., 2019). *DFR* had been already shown to be upregulated in the stem and leaf of tomato plants overexpressing *SlAN2* and *SlAN1* R2R3-MYBs (Kiferle et al., 2015). Interestingly, despite *FLS* was not found among upregulated DEGs in *PhAN4* HRCs, *LDOX*, a known bi-functional enzyme being able not only the conversion of leucoanthocyanidins in anthocyanidins but also to catalyze the formation of flavonols, resulted to be differentially upregulated in *PhAN4* HRCs. This may explain the accumulation of quercetin, kaempferol derivatives, and myricetin together with anthocyanidins. The flavonol biosynthetic activity of the upregulated *LDOX* is counterbalanced by the upregulated *DFR*. In tomatoes, DFR has a substantial preference for dihydromyricetin, which can be also derived by dihydrokaempferol and dihydroquercetin by F3′5′H activity. Interestingly, *DilFl* a tomato homolog of cytochrome b5 of *P. hybrida*, where it is essential for full activity of F3′5′H, was found to be upregulated in *PhAN4* HRCs. These findings may explain the differential accumulation of delphinidin and petunidin compared to pelargonidin, malvidin, and cyanidin (the latter was not detected at all). Among late biosynthetic genes, upregulation of *ANS* had been already shown upon *SlAN2* over-expression and *Del/Ros* expression in tomatoes. *OMT* and *AAT* transferases have already been described to be targets of a MYB in tomatoes, as well (Zhang et al., 2019). Their upregulation in *PhAN4* HRCs, together with that of *GST, PH3, OMT, 3UFGT, RT*, and *AAT* may explain the accumulation of the glycosylated and acylated forms of anthocyanidins. In particular, the function of the upregulated *PH3* (Solyc10g084380.1.1) in *PhAN4* HRC is rather to be associated with anthocyanin biosynthesis boosting than with acidification of pH, as tomato lacks *PH1* and *PH5* (i.e., the genes encoding the vacuolar P-ATPases which cooperate with PH3 and PH4 in determining flower color by hyperacidification of petal cell vacuoles in *Petunia*). Nevertheless, preliminary evaluation of pH of *PhAN4* and control HRC homogenates (Verweij et al., 2008) does not allow to exclude that the acidification function may occur (Supplementary Figure 5), given that pH differences between *PhAN4* and control HRC seem to be in accordance with variations recorded between vacuolar P-ATPases-defective mutants and wild type *Petunia hybrida* (Faraco et al., 2014). The upregulation of anthocyanin biosynthesis-relevant transcription factors further expands the influence of *PhAN4* MYB in tomato gene expression regulation. Among those, the upregulation of *SlAN1*, *SlAN2*, and *WD40* are coherent with already proposed models of anthocyanin biosynthesis regulation (Koes et al., 2005; Liu et al., 2018).

**TABLE 2:** Summary of results from the main research on tomato plants enrichment with anthocyanins obtained by both conventional breeding and genetic engineering approaches.

Tomato line	Origin	Main overexpressed genes	Main anthocyanins detected	Anthocyanin concentration	References
V118	Breeding	–	Pet-3-(p-coumaryl)-rut-5-glc; Pet-3-caeoyl-rut-5-glc; Mal-3(p-coumaryl)-rut-5-glc	50.18 mg 100 g^–1^ DW 9.04 mg 100 g^–1^ DW 13.09 mg 100 g^–1^ DW	Li et al., 2011
*Aft/Aft* × *atv/atv*	Breeding	–	Pet-3-(p-coumaroyl)-rut-5-glc; Del-3-rut	Peel: 116.11 mg 100 g^–1^ FW	Mes et al., 2008
Sun Black (*Aft/Aft* × *atv/atv*)	Breeding	–	Pet-3-(*trans*-p-coumaroyl)-rut-5-glc; Mal-3-(*trans*-p-coumaroyl)-rut-5-glc	More than 1 mg g^–1^ DW	Mazzucato et al., 2013
Blue Japan Indigo tomato (*Aft/Aft* × *atv/atv*)	Breeding	—	Pet + p-coumaroyl + rut + glyc; Mal + p-coumaroyl + rut; Del	Peel: 17 mg g^–1^ DW Pulp: 0.1 mg g^–1^ DW	Ooe et al., 2016
*Aft/Aft* × *atv/atv* × *hp2/hp2*	Breeding	–	Pet-(p-coumaroyl)-rut-hex; Del-3-(p-coumaroyl)-rut-glyc; Pet-(p-coumaroyl)-rut-hex; Pet-3-(caffeoyl)-rut-5-glyc; Mal-3-(p-coumaroyl)-rut-5-glyc; Cya-3-*O*-rut;	Peel: 90.91 mg 100 g^–1^ FW	da Silva Souza et al., 2020
*ANT1 from S. chilense*	Genetic engineering	*CHS, DFR, 3-GT, 5-GT, GST, ANP*	Del-3-rut-5-glc; Del-3-(p-coumaroyl)-rut-glyc; Del-3-(caffeoyl)-rut-5-glyc; Pet-3-rut-5-glc; Pet-3-(p-coumaroyl)-rut-5-glyc; Pet-3-(caffeoyl)-rut-5-glyc; Mal-3-rut-5-glc; Mal-3-(p-coumaryl)-rut-5-glyc; Mal-3-(caffeoyl)-rut-5-glyc	3.574 mg g^–1^ FW	Mathews et al., 2003
*Del/Ros1*	Genetic engineering	*PAL, C3H, CHI, F3′5′H, DFR, ANS, 3-GT, 5-GT, RT, AAC, GST, ANP*	Pet-3-(*trans*-p-coumaroyl)-rut-5-glc; Del-3-(*trans*-p-coumaroyl)-rut-5-glc; Pet-3-(feruloyl)-rut-5-glc; Del-3-(feruloyl)-rut-5-glc	2.835 ± 0.456 mg g^–1^ FW	Butelli et al., 2008
*Del/Ros1*	Genetic engineering	–	Del-3-(*trans*-p-coumaroyl)-rut-5-glc; Pet-3-(*trans*-p-coumaroyl)-rut-5-glc; Mal-3-(p-coumaroyl)-rut-5-glc; Mal-3-(feruloyl)-rut-5-glc	Peel: 5.1 ± 0.5 g kg^–1^ DW Flesh: 5.8 ± 0.3 g kg^–1^ DW Whole fruit: 5.2 ± 0.5 g Peo-3-glc equivalent kg^–1^ DW, or 0.5% of DW	Su et al., 2016
*ANT1 from S. lycopersicum*	Genetic engineering	*CHI, F3H, DFR, ANS, 3-GT*	Pet Mal Del	–	Schreiber et al., 2012
*SlANT1* and *SlAN2*	Genetic engineering	*SlAN2, SlANT1, SlAN1, SlAN11, SlJAF13, SlDFR*	–	–	Kiferle et al., 2015
*Del/Ros1* × *AtMYB12*	Genetic engineering	*PAL, 4CL, CHS, CHI, F3H, FLS, DFR, ANP, 3-GT, C3H*	Del-3-(*trans*-p-coumaroyl)-rut-5-glc; Pet-3-(*trans*-p-coumaroyl)-rut-5-glc; Pet-3-(feruloyl)-rut-5-glc; Mal-3-(p-coumaroyl)-rut-5-glc	1.154 ± 0.011 mg g^–1^ FW 2.857 ± 0.218 mg g^–1^ FW 0.922 ± 0.102 mg g^–1^ FW 0.598 ± 0.011 mg g^–1^ FW	Zhang et al., 2015
*SlMYB75*	Genetic engineering	*PAL, CHI, CHS, AAC, ANS, 3-RT, LDOX*	–	∼2 mg g^–1^ FW	Jian et al., 2019
*PhAN4*	Genetic engineering (transformed tomato hairy root cultures)	*CHS, CHI, F3′5′H, F3H LDOX, DFR, ANS, 3-GT, RT, OMT, AAT, ANP, GST*	Pet-3-(feruloyl)-rut-5-glc; Pet-3-(p-coumaroyl)-rut-5-glc; Pel-3glc; Mal-3-(4-coumaroyl)-rut-5-glc; Del-3-(p-coumaroyl)-rut-5-glc; Del-3-5-glc	37 μg g^–1^ DW	

*The main overexpressed genes and anthocyanins content are shown where available.*

*Del, delphinidin; Pet, petunidin; Mal, malvidin; Cya, cyanidin; Peo, peonidin; Pel, pelargonidin; rut, rutinoside; glc, glucoside; glyc, glycoside; hex, hexoside; FW, fresh weight; DW, dry weight.*

Interestingly, the *PhAN4*-related transcriptomic enhancement was found to involve more than 30 genes specifically related to response to biotic/abiotic and oxidative stress, as well. Indeed, GOE analysis was associated with proteinase inhibitors, beta-glucosidases, glucanases, glycosylases, xylan acetylases, resistance proteins, and other classes of enzymes that are players of defense against pathogens and pests and of response to abiotic stresses specifically associated with wounding, cold, heat, drought, hypoxia, and to UV exposure in tomato (Fan et al., 2020). Among those, the upregulated beta-glucosidases, together with the dirigent proteins and the monoterpenoid and sesquiterpenoid synthases that were found in the present work, are involved in the formation of required intermediates for cell wall lignification. The positive regulation of trichome birefringence-like protein mediating xylan acetylation also is another tool in possible protection against environmental stresses among which there is cold and excess of minerals in the soil. Acetylation of wall polymers is, indeed, vital for plant growth and adaptation to various environments, and is required for the structural integrity of the leaf surface exerting a global impact on plant stress responses (Gao et al., 2017). In relation to the positive regulation of beta-1,3-glucanases, beta-glucosidases, and xylan acetylases, together with expansions, it has to be added that these genes are related to fruit softening or improved emission of volatiles, as well (Tzin et al., 2015; Li P. et al., 2020). In this sense, the positive regulation of chlorophyll a/b binding protein (that is normally upregulated during fruit ripening) and of one gene related to volatile compounds biosynthesis (*chorismate mutase*) was found in *PhAN4* HRCs. At the same time, negative regulation of other genes, which down expression is involved in the extended shelf life of fruits, was found. The evaluation of these aspects might be important in view of the development/design of whole transgenic tomato plants in relation to the quality traits of fruits.

One player of ROS detoxification and regulator of the redox signaling network of tomato, plastidial thioredoxin Y2, was found to be highly upregulated, as well. Moreover, the analysis demonstrated the downregulation of violaxanthin de-epoxidase (*VDE*) and carotenoid isomerase (*CRTISO*), two genes involved in carotenoid biosynthesis. Despite being involved in the photo-inhibition of the PSII, suppression of VDE can induce, at the same time, an accumulation of fucoxanthin that functions as an efficient anti-oxidant in anoxia conditions (Mikami and Hosokawa, 2013). The downregulation of carotenoid isomerase was demonstrated to induce an accumulation of zeta-carotene and *cis*-prolycopene in tomato fruits (Isaacson et al., 2002; Pinheiro et al., 2019), both elevating and modifying carotenoid profiles toward more bioavailable forms compared to wild-type (Cooperstone et al., 2015). Therefore, the ROS counteracting potential of *PhAN4* HRC material may be related not only to anthocyanins but also to other accumulating anti-oxidant specialized metabolites.

In view of the future development of natural anti-oxidants produced by engineered plants and possibly administered with the diet, and to define whether the significantly higher DPPH antioxidant capacity of specialized metabolites accumulating in *PhAN4* HRCs may be efficiently maintained after ionizing radiation, we exposed lyophilized biomass from AN4-1 HRC to high dose ^60^Co gamma radiation. Gamma rays are a component of cosmic ionizing radiation and induce the formation of free radical species (i.e., paramagnetic species). When carried out in an air atmosphere, gamma rays induce the formation of ROS, such as superoxide, peroxide, and hydroxyl radicals, that are responsible for several oxidative processes in biological systems (Marcu et al., 2013b; Schreurs et al., 2016). Gamma rays are perfect ROS inducers both directly through water radiolysis, and indirectly *via* the activation of a broad range of signaling processes (e.g., damages to the mitochondria or cell microenvironment) (Riley, 1994; Azzam et al., 2012; Buttarelli et al., 2019). ESR Spectroscopy was used as a sensitive tool to identify the entity of the paramagnetic species generated in the HCRs samples after gamma irradiation (Aleksieva et al., 2009; Hawkins and Davies, 2014; D’Errico et al., 2018). ESR analyses revealed a significant difference in the overall amounts of radical species accumulated in the two HRC molecular backgrounds. At the same absorbed dose, control generates a very intense peroxyl radical signal, while a considerably lower level of the singlet intensity is generated in AN4-1. This result reveals that the molecular set formed upon *PhAN4* expression provides very effective free radical scavengers efficiently counteracting oxidative stress upon gamma radiation.

The UV-VIS analysis allowed us to confirm the results obtained by metabolomic data and to characterize the effect of ionizing radiation on the antioxidant compounds. Gamma irradiation, *via* releasing free radicals in solution, may alter these plant constituents (Lateef and Al-Nimer, 2009; Lalande et al., 2019). Compared to the substantially unchanged spectra of AN4-1, the absorbance decrease of the 320 nm peak (i.e., flavonols, hydroxycinnamic acids, tannins, flavonols, and anthocyanins associated to caffeic and coumaric acids) controls upon irradiation, suggests that the anthocyanins accumulated in *PhAN4* HRCs are particularly stable to gamma high absorbed dose and probably have protective effects on other biomolecules. Indirectly, this result shows that these molecules may give a major contribution to the ROS buffering capacity under radiation shown by ESR and to the overall antioxidant potential. As a confirmation of the possible significance of this result *in vivo*, it was reported that, even at higher absorbed doses than that used in this work, X rays (e.g., another type of ionizing radiation) leave the level of flavonoids in an aqueous solution unchanged due to the radiolysis-mediated formation of depsides that, in addition, maintain good anti-oxidant properties, as well (Kozlowski et al., 2007; Lateef and Al-Nimer, 2009).

For proteins to be functional within a cell requires coordinated folding processes to obtain a correct 3D shape. Disruptions to protein folding, that can occur in space due to ionizing radiation, can have profound biological implications for all organisms, including plant cells, leading to dysfunctions (Blanco et al., 2018; Lalande et al., 2019). For this reason, investigations were conducted through photoluminescence analysis on HRCs’ total soluble protein extracts. When a protein is exposed to the wavelength of 280 nm, mainly the tryptophan and tyrosine residues get excited, which would reflect upon its tertiary structure (Yang H. et al., 2015). The maximum and emission peak position reflects upon secondary, tertiary, and quaternary structures (Hilaire et al., 2017). Moreover, the extent of protein oxidation is measured by determining the loss of specific tryptophan fluorescence. The emission spectra of HRCs showed an overall decrease in maximum emission intensities only in the case of irradiated controls, revealing that the structure of soluble proteins is partially lost upon irradiation in those samples. This result demonstrates a low capacity of proteins of control HRCs to counteract unfolding, binding to hydrophobic pockets, and aggregation, witnessed by the loss of fluorescence due to the burial of tryptophan residues after radiation. In particular, the Δ% photoluminescence intensity/mass units after and before irradiation indicated that soluble proteins from control undergo a doubled oxidative stress compared to AN4-1 samples. Being polyphenols emitting at 280 and 320 nm extracted by PBS together with total soluble proteins (anthocyanins are not efficiently extracted, Supplementary Figure 4), the contribution of these classes of molecules may be hypothesized in stabilizing proteins, as proofs of the interaction of anthocyanins with proteins seem to suggest (Sui et al., 2018). Possible additional contributions of enzymatic players to mitigation of misfolding and aggregation *in vivo* may be the object of future studies.

In conclusion, plant biotechnology methods may thus be exploited for the generation of plants capable of dealing with harsh conditions such as those typical of space outposts. MicroTom HRC allowed to rapidly test *PhAN4* expression effects on tomato cells, possibly opening the way to apply to the engineering of whole plants able to perform in a suitable and predictable manner in those environments.

## Data Availability Statement

The datasets presented in this study can be found in online repositories. The names of the repository/repositories and accession number(s) can be found below: https://dataview.ncbi.nlm.nih.gov/object/PRJNA794337?reviewer=estprpllasrt3f3libdoij8rco.

## Author Contributions

SM planned and designed the project, undertook tomato transformation experiments, PCR screening and maintenance of HRCs, and sample preparation for subsequent analysis, as well as wrote the manuscript. RP handled maintenance of HRCs, contributed to sample preparation and transcriptomic data retrieving and interpretation, and contributed to the writing of the manuscript and the preparation of figures. AB performed sequencing read mapping, identification of DEGs and functional annotation, and enrichment pathway analysis of DEGs. HP undertook cDNA library construction and sequencing for transcriptomic analysis. MB contributed to the identification of DEGs and functional annotation of genes. AC and IS performed gamma irradiation experiments, ESR, UV-VIS, and photoluminescence analysis and contributed to writing the manuscript. GD and OD performed MS analysis, anthocyanin identification/quantification, and contributed to writing the manuscript. FP conducted the qPCR analysis. AD and PD performed the Trolox assay and the quantification of total polyphenol content and contributed to writing the manuscript. FQ, RK, and CS assembled and kindly provided the PhAN4 constructs used in this work. ElB contributed to the maintenance of HRCs. EuB reviewed and contributed to the writing of the manuscript. All authors contributed to the article and approved the submitted version.

## Conflict of Interest

The authors declare that the research was conducted in the absence of any commercial or financial relationships that could be construed as a potential conflict of interest.

## Publisher’s Note

All claims expressed in this article are solely those of the authors and do not necessarily represent those of their affiliated organizations, or those of the publisher, the editors and the reviewers. Any product that may be evaluated in this article, or claim that may be made by its manufacturer, is not guaranteed or endorsed by the publisher.

## Footnotes

1
https://github.com/KoesGroup/Snakemake_hisat-DESeq

2
http://broadinstitute.github.io/picard/

3
https://ggplot2.tidyverse.org/

## Supplementary Material

Supplementary Table 1 Primer identification (id.) as described in “Materials and Methods” for HRCs PCR screening (for, forward; rev, reverse).

Supplementary File 1 List of figures representing the level of different phenolic compounds and their derivatives in control and *PhAN4* HRCs.

Supplementary File 2 List of figures representing the level of different flavonoids in control and PhAN4 HRCs.

Supplementary File 3 Complete list of DEGs.

Supplementary File 4 GOE analysis results.
