# Supplementary material for: Modifying Anthocyanins Biosynthesis in Tomato Hairy Roots: A Test Bed for Plant Resistance to Ionizing Radiation and Antioxidant Properties in Space
Source: Front Plant Sci. 2022 Feb 24;13:830931. doi: 10.3389/fpls.2022.830931 (PMC8909381; doi:10.3389/fpls.2022.830931)

## Slide 1
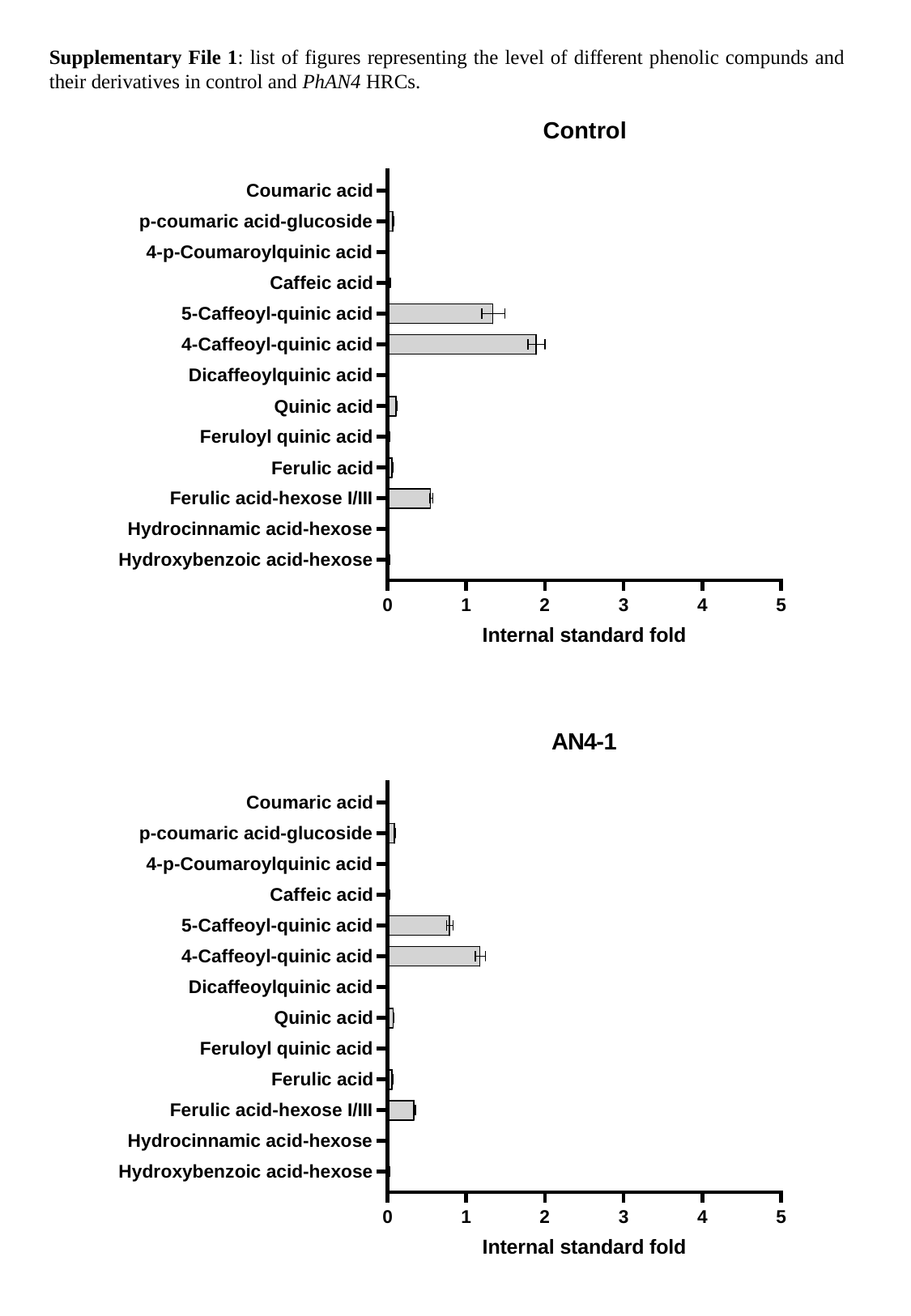

Supplementary File 1: list of figures representing the level of different phenolic compunds and their derivatives in control and PhAN4 HRCs.

## Slide 2
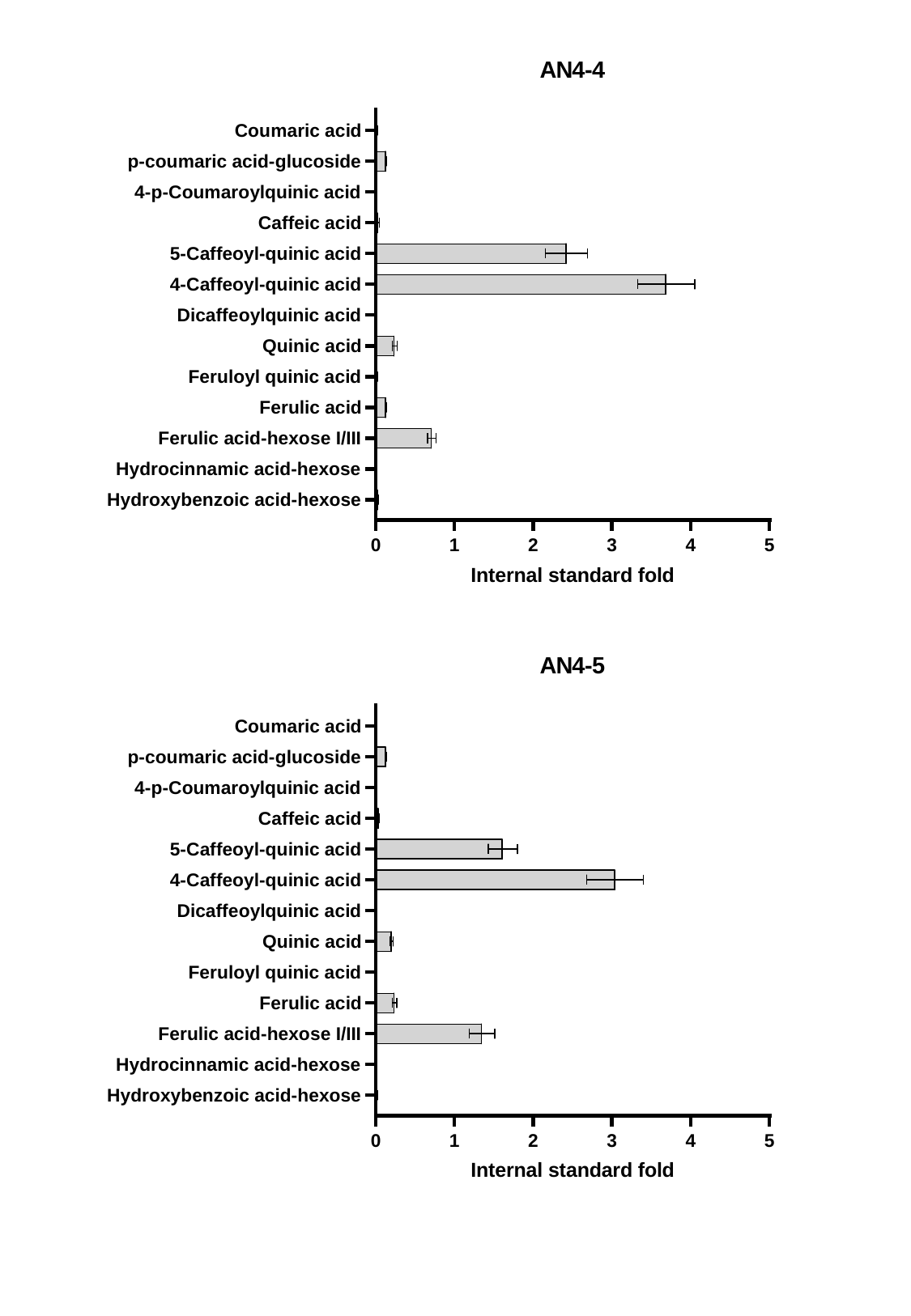

## Slide 3
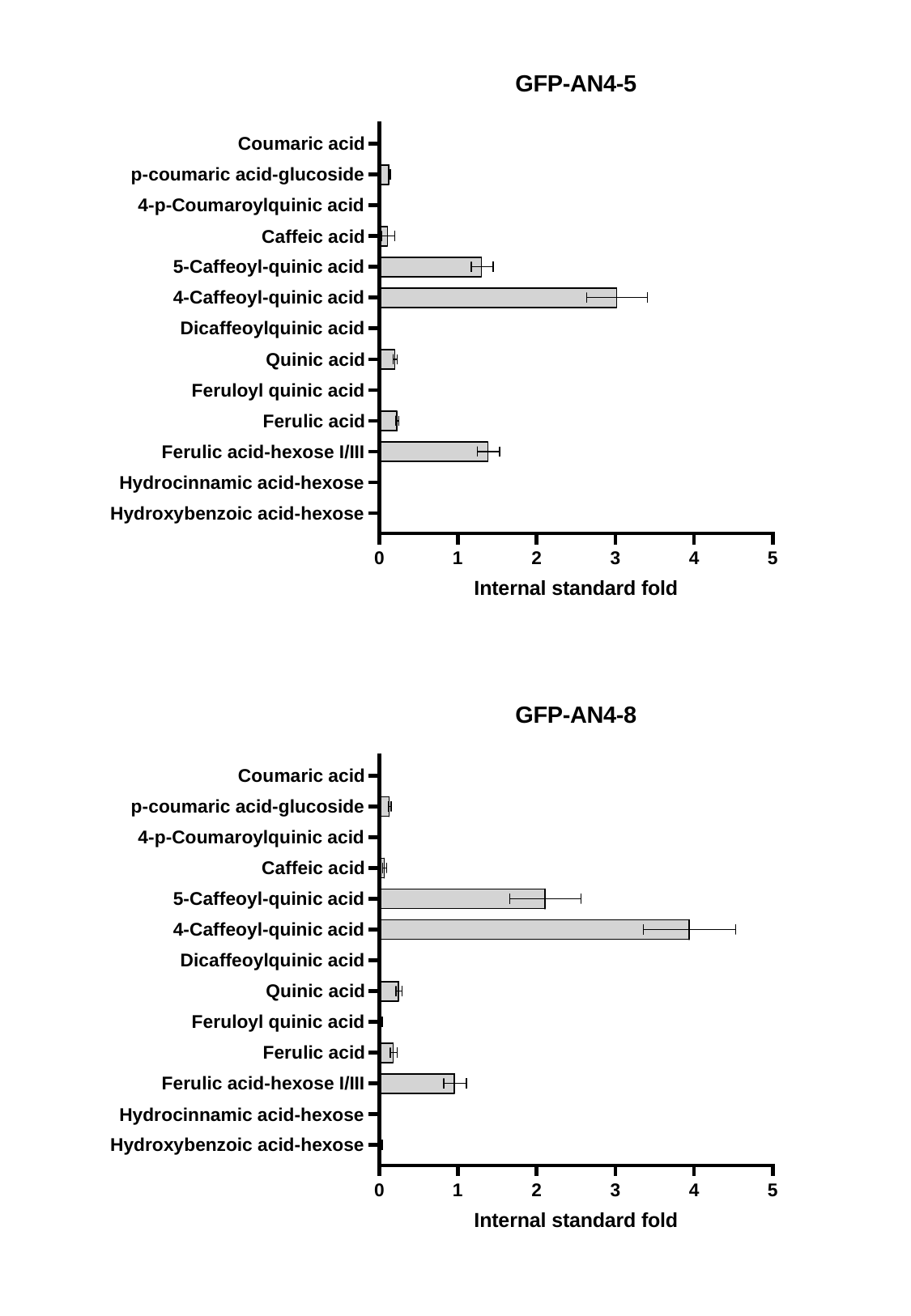

## Slide 4
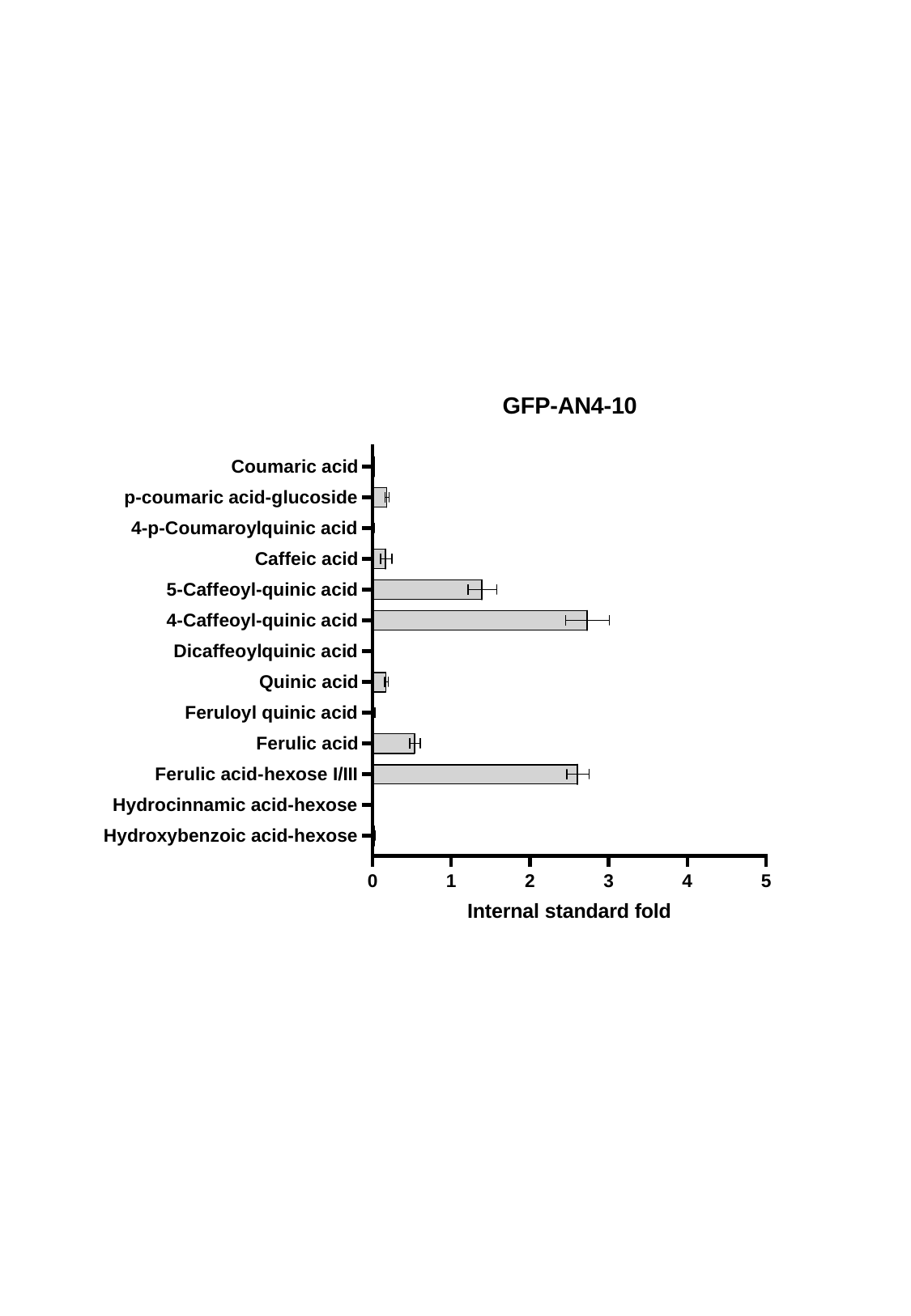

Supplement: Supplementary File 1 — List of figures representing the level of different phenolic compounds and their derivatives in control and PhAN4 HRCs. [file Presentation_2.PPTX]
