# Supplementary material for: Modifying Anthocyanins Biosynthesis in Tomato Hairy Roots: A Test Bed for Plant Resistance to Ionizing Radiation and Antioxidant Properties in Space
Source: Front Plant Sci. 2022 Feb 24;13:830931. doi: 10.3389/fpls.2022.830931 (PMC8909381; doi:10.3389/fpls.2022.830931)
Supplement: Supplementary File 3 — Complete list of DEGs. [file Presentation_1.PPTX]

## Slide 1
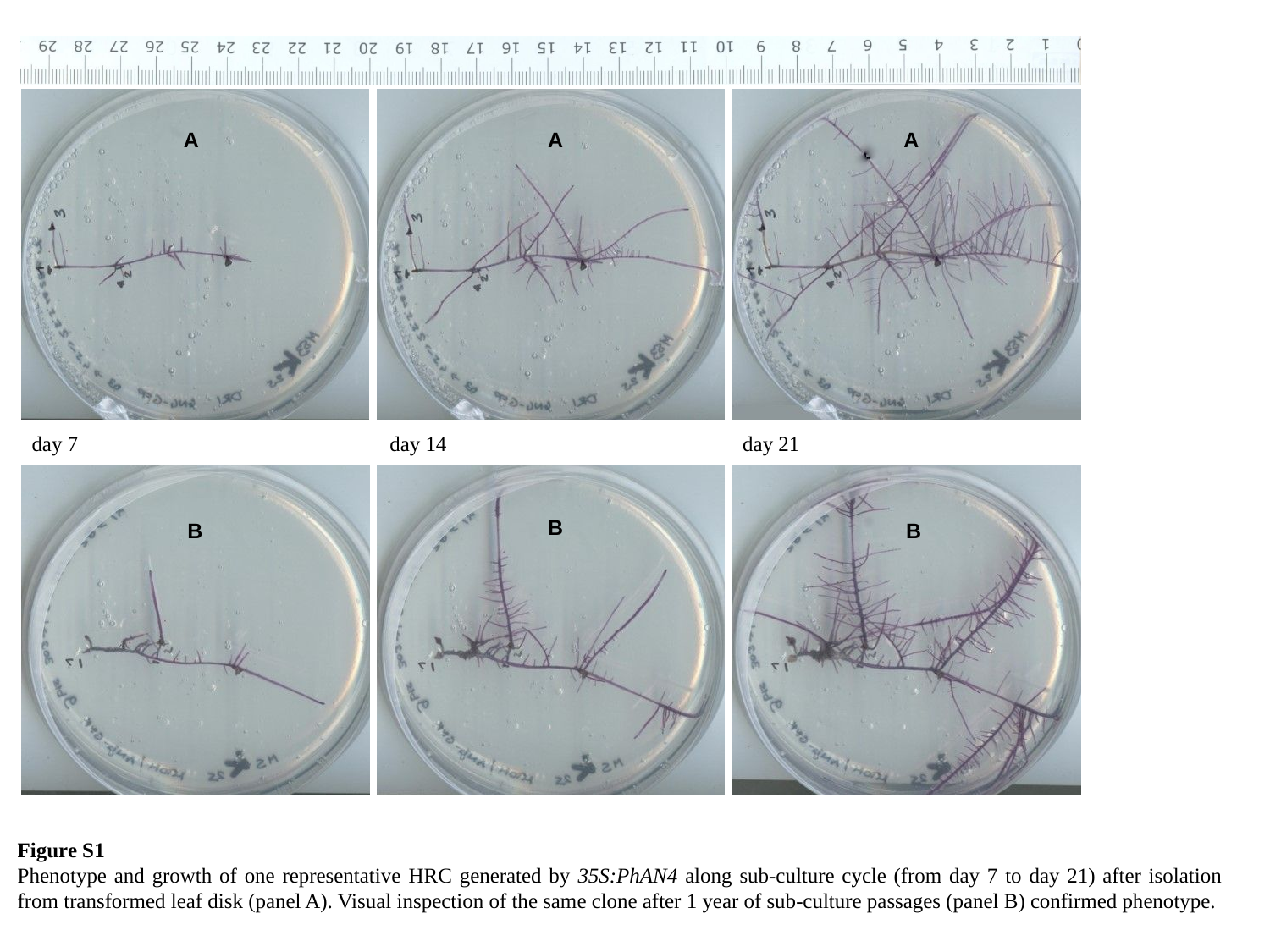

A
A
A
day 7 day 14 day 21
B
B
B
Figure S1
Phenotype and growth of one representative HRC generated by 35S:PhAN4 along sub-culture cycle (from day 7 to day 21) after isolation from transformed leaf disk (panel A). Visual inspection of the same clone after 1 year of sub-culture passages (panel B) confirmed phenotype.

## Slide 2
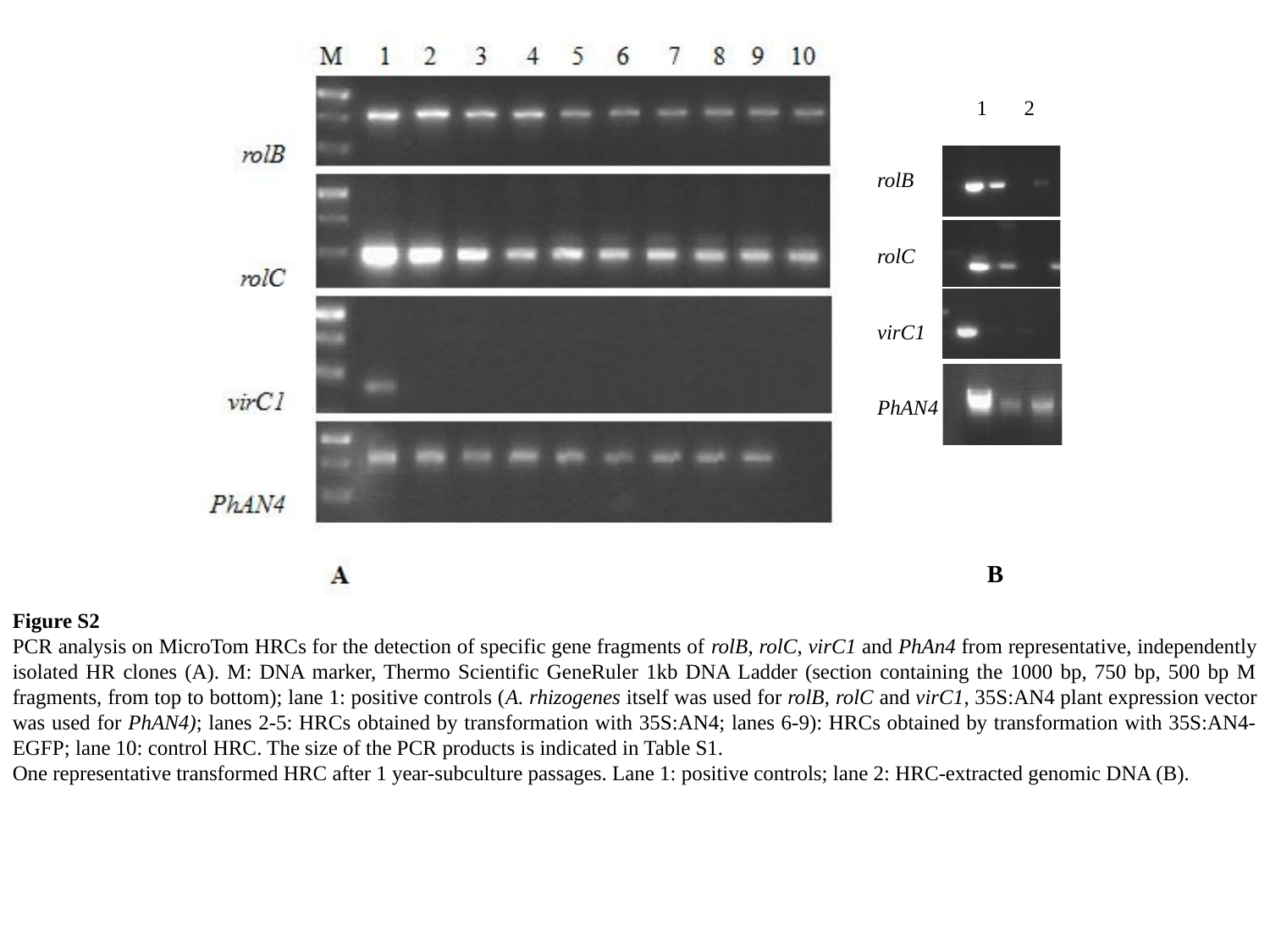

1 2
rolB
rolC
virC1
PhAN4
B
Figure S2
PCR analysis on MicroTom HRCs for the detection of specific gene fragments of rolB, rolC, virC1 and PhAn4 from representative, independently isolated HR clones (A). M: DNA marker, Thermo Scientific GeneRuler 1kb DNA Ladder (section containing the 1000 bp, 750 bp, 500 bp M fragments, from top to bottom); lane 1: positive controls (A. rhizogenes itself was used for rolB, rolC and virC1, 35S:AN4 plant expression vector was used for PhAN4); lanes 2-5: HRCs obtained by transformation with 35S:AN4; lanes 6-9): HRCs obtained by transformation with 35S:AN4-EGFP; lane 10: control HRC. The size of the PCR products is indicated in Table S1.
One representative transformed HRC after 1 year-subculture passages. Lane 1: positive controls; lane 2: HRC-extracted genomic DNA (B).

## Slide 3
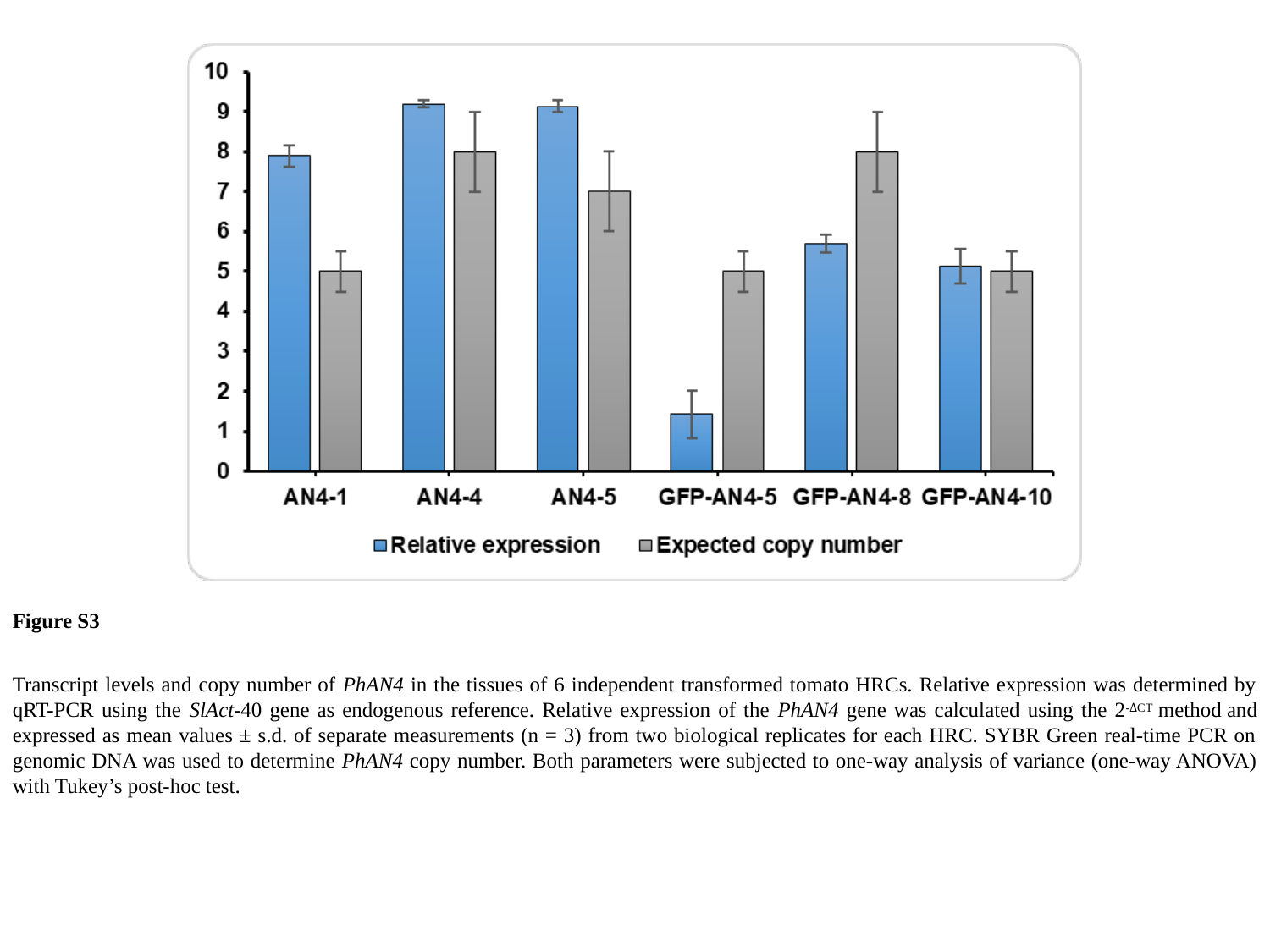

Figure S3
Transcript levels and copy number of PhAN4 in the tissues of 6 independent transformed tomato HRCs. Relative expression was determined by qRT-PCR using the SlAct-40 gene as endogenous reference. Relative expression of the PhAN4 gene was calculated using the 2-ΔCT method and expressed as mean values ± s.d. of separate measurements (n = 3) from two biological replicates for each HRC. SYBR Green real-time PCR on genomic DNA was used to determine PhAN4 copy number. Both parameters were subjected to one-way analysis of variance (one-way ANOVA) with Tukey’s post-hoc test.

## Slide 4
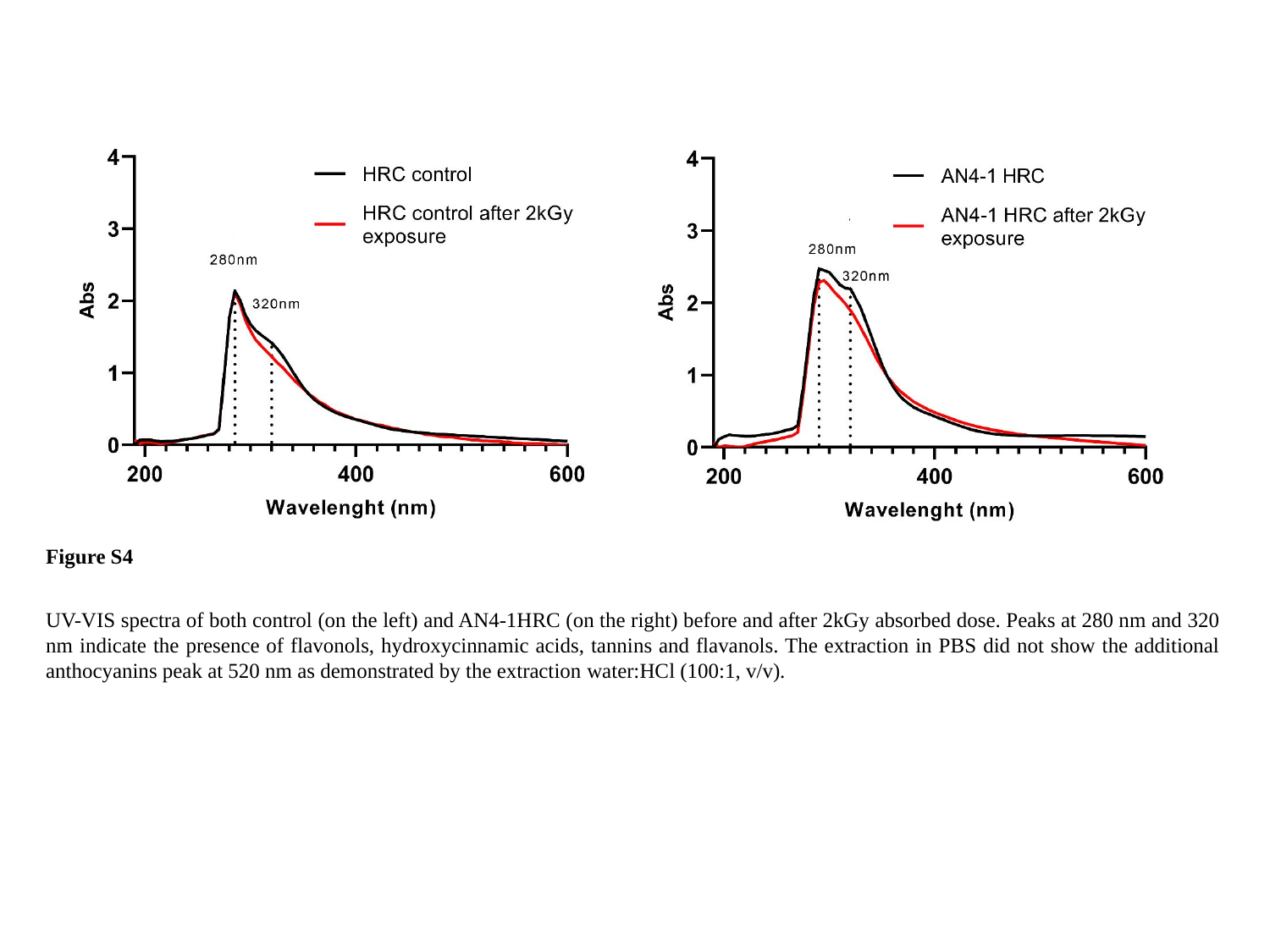

Figure S4
UV-VIS spectra of both control (on the left) and AN4-1HRC (on the right) before and after 2kGy absorbed dose. Peaks at 280 nm and 320 nm indicate the presence of flavonols, hydroxycinnamic acids, tannins and flavanols. The extraction in PBS did not show the additional anthocyanins peak at 520 nm as demonstrated by the extraction water:HCl (100:1, v/v).

## Slide 5
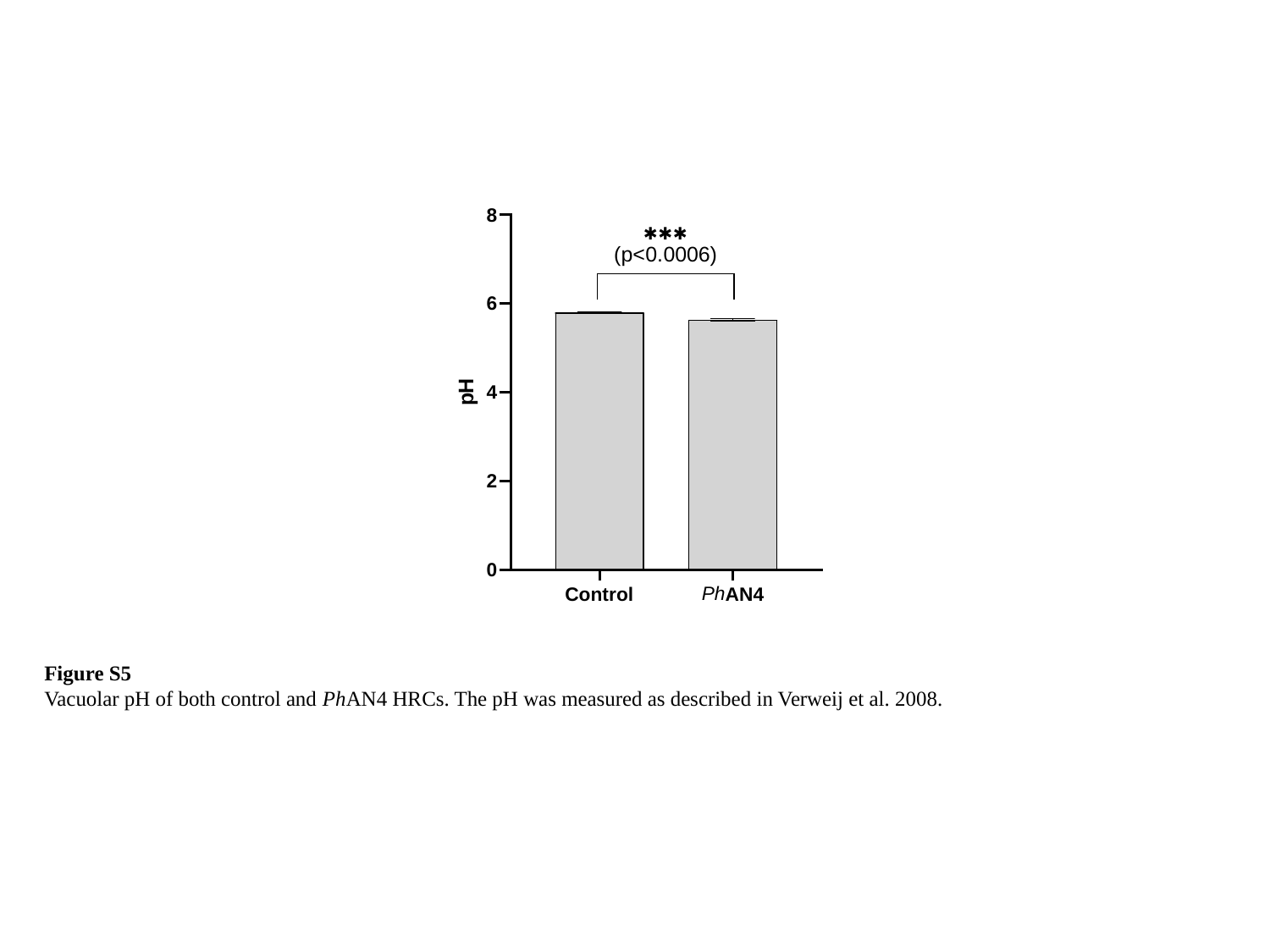

Figure S5
Vacuolar pH of both control and PhAN4 HRCs. The pH was measured as described in Verweij et al. 2008.
