# Supplementary material for: Modifying Anthocyanins Biosynthesis in Tomato Hairy Roots: A Test Bed for Plant Resistance to Ionizing Radiation and Antioxidant Properties in Space
Source: Front Plant Sci. 2022 Feb 24;13:830931. doi: 10.3389/fpls.2022.830931 (PMC8909381; doi:10.3389/fpls.2022.830931)
Supplement: Supplementary file 6 [file Table_3.DOCX]

| PCR Oligonucleotide i.d. | Sequence 5’-3’ | Amplified sequence lenght |
| --- | --- | --- |
| RolB for | 5’ - ATG GAT CCC AAA TTG CTA TTC CTT CCA CGA - 3’ | 776 bp |
| RolB rev | 5’ - TTA GGC TTC TTT CTT CAG GTT TAC TGC AGC - 3’ |  |
| RolC for | 5’ - TGT GAC AAG CAG CGA TGA GC - 3’ | 487 bp |
| RolC rev | 5’ - GAT TGC AAA CTT GCA CTC GC - 3’ |  |
| VirC1 for | 5’ - AAT GCG TCT CTC TCG TGC AT - 3’ | 425 bp |
| VirC1 rev | 5’ - AAA CCG ACC ACT AAC GCG AT - 3’ |  |
| PhAN4 for | 5’ - ATG AAA ACT TCT GTT TTT ACG TCG TC - 3’ | 768 bp |
| PhAN4rev | 5’ - TTA TAG TAA TTC CCA GAG GTC AGC ATC - 3’ |  |
| AN4_FW | 5’ -GGGGACAAGTTTGTACAAAAAAGCAGGCTGGATCCGAAAAAAATGGGTA - 3’ |  |
| AN4_RV | 5’ - GGGGACCACTTTGTACAAGAAAGCTGGGTTCAGTGCAGTTCGTTCAACAA - 3’ |  |
|  |  |  |
|  |  |  |
| qPCR Oligonucleotide i.d. | Sequence 5’-3’ |  |
| SlACT41for | 5’ - GCTCTTGACTATGAACAGGAAC - 3’ | 126 bp |
| SlACT41 rev | 5’ - AAGGACCTCAGGACACCG - 3’ |  |
| q PhAN4 for | 5’ - GCCGAAGTCGTGTCCAGAGACG - 3’ | 105 bp |
| q PhAN4 rev | 5’ - AAATGCTGAAGTGCTTTCAGTTG - 3’ |  |

**Supplementary Table**: Primer identification (id.) as described in materials and methods for HRCs PCR screening (for: forward, rev: reverse).
